# Patient-Centered Prioritization of Health Care Processes for Multimorbidity

**DOI:** 10.1001/jamanetworkopen.2025.49693

**Published:** 2025-12-01

**Authors:** Linnaea Schuttner, Mariah Theis, Edwin Wong, Jonathan Staloff, Robert Brett McQueen, Karin Nelson, James D. Ralston, Laura Coyle, Scott Hagan, Tamara Schult, Traci Solt, Katherine Ritchey, Ann-Marie Rosland

**Affiliations:** Health Services Research & Development, VA Puget Sound Health Care System, Seattle, Washington (Schuttner, Theis, Wong, Staloff, Nelson); Department of Medicine, University of Washington, Seattle (Schuttner, Nelson, Hagan); Department of Health Systems and Population Health, School of Public Health, University of Washington, Seattle (Wong, Ralston); Department of Family Medicine, University of Washington, Seattle (Staloff); University of Colorado School of Pharmacy, Aurora (McQueen); Kaiser Permanente Washington Health Research Institute, Seattle (Ralston); VA Center for Healthcare Evaluation Research and Promotion, VA Pittsburgh Healthcare System, Pittsburgh, Pennsylvania (Rosland); Department of Medicine, University of Pittsburgh, Pittsburgh, Pennsylvania (Rosland); Office of Primary Care, Veterans Health Administration, Washington, District of Columbia (Coyle, Solt); Office of Patient Centered Careand Cultural Transformation, Veterans Health Administration, Washington, District of Columbia (Schult); Office of Assistant Under Secretary for Health for Integrated Veterans Care, Washington, District of Columbia (Solt); Geriatrics Research, Education and Clinical Center, VA Puget Sound Health Care System, Seattle, Washington (Ritchey); Division of Geriatrics and Gerontology, University of Washington, Seattle (Ritchey)

## Abstract

**IMPORTANCE:**

Primary care provides a broad service range, including preventive screenings and chronic disease care. Busy clinicians frequently select which health care processes to address per visit based on time or perceived disease impact, especially for patients with multimorbidity. Little evidence exists for how to prioritize processes by patient-centered health goals.

**OBJECTIVE:**

To identify criteria for prioritizing health care processes for patients with multimorbidity and using these criteria, develop recommended priority rankings of health care processes aligned with patient-centered health goals.

**EVIDENCE REVIEW:**

This consensus statement was developed using multicriteria decision analysis, a group consensus method. Using purposeful sampling, 21 national subject matter experts were recruited across 10 sites, with input from patient and health system partners. Experts completed 2 electronic survey rounds. The first round was conducted February 26 to April 2, 2024, with experts weighting 12 criteria for health care processes by importance (0–100 scale, with higher score indicating more importance) for addressing 3 common domains of patient-centered health goals (mortality, symptom relief, physical function) for hypothetical patients with multimorbidity. The second round was conducted June 3 to July 26, 2024, with experts scoring 53 health care processes by criteria (1–9 scale, with higher scores indicating greater priority). Experts received an evidence summary (publications through January 19, 2023) underlying health care processes. Weights and scores were combined as weighted sums, converted to process rankings per goal. Monte Carlo simulation (5000 randomizations) was used to evaluate confidence in findings (described as percentage of iterations at rank).

**FINDINGS:**

A total of 20 experts participated (95% response rate). Survey 1 determined attributes important for decision-making. Seven criteria were selected: actionability, safety impact, completion ease, impact on high-prevalence health issues, evidence quality, long-term health outcome impact, and validity. The second survey used these criteria to rank health care processes by patient goals. Tobacco use screening was the highest priority health care process for all goals (function: total score, 6.9 [49% of iterations ranked as No. 1]; mortality: total score, 7.4 [27% of iterations ranked as No. 1]; symptoms: total score, 7.1 [44% of participants ranked as No. 1]). Rankings for remaining health care processes varied by goal.

**CONCLUSIONS:**

In this consensus statement, consideration of patient-centered health goals led to varied recommended decision-making criteria and prioritization of health care processes by subject matter experts. For patients with multimorbidity, these findings could inform systems evaluating health care process applications (eg, selection of screenings for patient-centered quality initiatives) or clinicians personalizing care delivery for patients (eg, catalyzing conversation about goals or efficiently addressing processes if goals are known).

## Introduction

Guideline-concordant preventive care and chronic disease management tasks, such as cancer screening or monitoring for diabetic nephropathy, are core components of primary care. These routine health care processes are often operationalized into care reminders and quality metrics within health care systems. These processes are important but numerous. One study estimated that fulfilling only preventive and chronic disease-based health care processes for a typical patient panel would take more than 9 hours daily per primary care practitioner (PCP) in a team-based care model, exceeding time commonly available.^1^ While many health care processes are completed by support staff, completion can be variable,^2^ and PCPs have an important role in ordering testing, discussing screening, and stewarding team resources for process completion. However, in time-limited settings, PCPs may defer or skip health care processes or process follow-up altogether.^3–5^ This is particularly true for patients with multimorbidity, who often have additional acute needs or psychosocial concerns to address, or patients for whom care guidelines or evidence may be less defined.^6,7^

To decide how to prioritize care, many PCPs default to prognosis or acuity,^7–9^ while health systems may prioritize disease-oriented outcomes or utilization.^10,11^ Shared agenda setting^12^ or using patient health outcome goals^11,13^ to personalize care plans can help to make decisions around health care processes more patient-centered, yet can be effortful if applied to all processes or patients. Prior studies have attempted to prioritize among some types of health care processes or for some patients,^14^ for example, recommended approaches to preventive health care processes to maximize population-level benefits.^15,16^ However, to our knowledge, no guidance exists to help prioritize decision-making involving patient health goals through a more systematic, efficient approach.

We had 2 objectives for this consensus statement. First, we aimed to clarify optimal criteria that PCPs or health system decision-makers could consider when choosing which health care processes to address for patients with multimorbidity, if processes are to be aligned to patient-centered health goals. Second, we aimed to provide recommendations on the priority ranking of routine health care processes, according to common health goals^17–19^ for patients with multimorbidity. These findings could provide a starting position for clinicians to make quicker decisions oriented to patient health goals within visits, catalyze clinician-patient conversations to guide downstream care, or help systems or policymakers take a more patient-centered approach to developing or evaluating existing health care processes.

## Methods

The protocol for this consensus statement was approved by the VA Puget Sound institutional review board with a waiver of participant written informed consent, as the study was qualified as minimal risk to participants. The protocol was not prospectively registered. This consensus statement is reported following the Accurate Consensus Reporting Document (ACCORD) guideline for consensus methods in biomedicine.^20^

### Overview

We used multicriteria decision analysis (MCDA) as a consensus approach,^21^ a group-based decision-making exercise permitting incorporation of multiple viewpoints and factors. MCDA has been successful in health-related decisional processes but has not previously been applied to these objectives, to our knowledge.^22–24^ Because MCDA enables integration of complex, even conflicting decisional factors,^25,26^ it lends to reconciling disparate perspectives, allowing synthesis of a systems perspective focused on population-level processes and the perspectives of individuals focused on personalized care. Compared with other consensus techniques (eg, Delphi panels), MCDA offers advantages in reproducibility and transparency, with potential reduced cognitive and motivational bias.^25,26^ MCDA has 4 basic steps (Figure): problem selection, selecting criteria most important to make decisions, evaluating items to be scored by criterion, then combining components mathematically for a solution.

We involved multiple participants to generate decisional criteria and priority ranking of health care processes, varying by contribution toward specific patient-centered health goals. We focused on 3 broad health goal domains common among patients with multimorbidity: mortality reduction, improving physical and cognitive function, and symptom and pain relief. These goal domains encompass many individual health outcome goals used to personalize care planning and provide goal-directed care.^17–19,27^ We focused on patients with multimorbidity (ie, ≥2 chronic diseases) and increased risk of adverse events, as these patients are likely to have other needs interfering with the completion of routine health care processes^28–30^ and to be better served by focusing on person-centered rather than disease-based health goals.^6^ Further details are provided in the eMethods in the Supplement.

### Participants

Three groups provided perspectives: subject matter experts (SMEs), patients advisors, and a health systems advisory board. SMEs were purposefully recruited with input from health system advisory partners, based on clinical, research, or leadership expertise. Enrollment targeted 15 to 21 SMEs.^31,32^ Recruitment was conducted via email; of 42 SMEs emailed, 21 (50%) enrolled. Participants were sent 2 rounds of an electronic Qualtrics survey. Round 1 was completed between February 26 and April 2, 2024; round 2, between June 3 and July 26, 2024. SME identities were anonymized and participants blinded to identities of other SMEs. In round 1,18 of 21 SMEs (86%) participated; in round 2,19 of 21 SMEs (90%) participated; overall, 20 of 21 SMEs (95%) participated in at least 1 round. SMEs were offered $45 for completing both rounds.

Patient advisory input was solicited through 2 sessions (in 2021 and 2023) with the VA Puget Sound Veteran Engagement Board, a panel of 6 to 10 diverse veterans representing the Veterans Health Administration (VHA) patient community, including individuals with multimorbidity, trained as research consultants and compensated by the institution. During 2 sessions of facilitated, online group discussions, the Veteran Engagement Board provided input on the patient-centered goal domains and decision criteria used within the survey of SMEs.

A health system advisory board also supported this consensus method, including national and local clinical operational leaders and organizational representatives (L.C., S.H., T. Schult, T. Solt, and K.R.). This board met virtually every 6 months for feedback during the study. The board provided discussion and voting-based feedback during group meetings and asynchronous, individual feedback on the study design and survey materials, including SME recruitment, initial and round 1 to 2 decision criteria selection, health care process selection and construct development, and survey design.

### MCDA

We began with a list of 28 potential criteria based on literature,^14,19,33^'^39^ discussed with patient and health system advisors. Twelve criteria were selected, aligned with MCDA best practices.^25^ We reviewed all 373 health care processes used in the VHA in 2023 for preventive care, screening, or chronic disease management with potential relevance to a patient visit with a frontline PCP. After feedback from patient and health system advisory boards and consolidation into constructs (eg, diabetic glycemic control, rather than describing an A_1c_ target), 53 health care processes were included in MCDA.

The first survey objective was selecting and weighting decisional criteria to rank health processes. Each SME received 1 of 3 survey versions, differing by the patient-centered goal domains: mortality (extending life), improving or maintaining physical and cognitive functioning, and symptom reduction, including pain and mental-health symptoms. Goal domains were selected based on literature^8,17,40–42^ and patient advisory group input. SMEs were asked to weigh potential criteria importance by allocating up to 100 points (more points for greater import) for aligning care to the specific health goal. SMEs were asked to consider patients with multimorbidity in responses. However, if helpful, they were asked to consider hypothetical veterans with multimorbidity as at least 2 of the following conditions: diabetes, hypertension, chronic kidney disease, and depression, and at least 75% risk of mortality or hospitalization in the next 3 months using a validated VHA risk-prediction score.^43^ This combination was selected as representing common, prevalent diseases clustering among veterans at high risk of adverse outcomes^44^ likely to have interactions in disease treatment plans^45^ (and likely to receive more benefit from focusing on patient-centered goal domains). SMEs could also add free-text alternative criteria and assign additional points. We normalized responses toa 100-point scale and calculated mean and SDs per criterion, per goal. Free-text responses were reviewed by 2 study members (L.S. and M.T.) according to principles governing MCDA criteria: complete, nonredundant, nonoverlapping, and assessable independently.^46^ We reviewed results with the health system advisory board, and for survey burden, selected only the top 4 criteria per health goal. With overlap between multiple top 4 criteria between goals, this led toa final 7 criteria from the original 12 for survey 2.

The second survey priority-ranked health care processes by scoring according to selected criteria from survey 1. We included the 53 health care processes for scoring by 7 final criteria from survey 1. For this, SMEs were provided an evidence summary for health care processes. Health care processes were divided across SMEs to reduce survey burden; each SME was randomly assigned up to 11 processes. SMEs scored health care processes on a 10-point Likert scale from worst- to best-performing per criterion. To reduce response-order effect, presentation order of health care processes and criteria was randomized.

The survey results were then combined using weighted sums. The mean criteria importance-weight per goal was multiplied by the health care process score for that criterion.^25^ These values summed for each health care process, with a higher total (10.0 maximum) representing better alignment with a health goal. Sums were translated to priority rankings of health care processes, varied by health goal.

To determine potential uncertainty in weighting and health care process scoring within MCDA,^47^ we incorporated Monte Carlo simulation modeling for all criteria weights and for the top and bottom 15 ranking (total sums) health care processes.^48^ We present uncertainty as the proportion of total iterations resulting in a rank, and plus or minus 1 from the original rank. Higher proportions reflect lower uncertainty.

### Evidence Summary

To assist SMEs, we developed an evidence summary for the health care processes (eTable 1 in the Supplement). We searched disease-based and preventive care clinical practice guidelines (CPGs) through January 19,2023, related to the health care processes. Twenty relevant CPGs were reviewed and summarized into an evidence matrix provided to participants, providing a description of the health care process, target population, disease prevalence, and a summary of clinical practice guidelines and evidence grade.

## Results

### SME Demographics

A total of 20 SMEs participated across 9 VHA facilities and 10 university affiliates. Five SMEs (25%) were high-level leadership (eg, division chair) in VHA or academic affiliates, all were clinicians, and most (17 of 21 SMEs invited [85%]) had significant relevant research experience (Table 1).

### Criteria for Deciding Which Health Care Processes to Address

Criteria importance varied relative to the patient-centered goal domain of interest (Table 2). Across all goals, if a health care process was directly actionable, it was weighted as the most important criterion for evaluating health care processes (mortality: mean [SD], 25.3 [37.6]; function: mean [SD], 16.4 [5.5]; symptom relief: mean [SD], 15.2 [5.2]). In contrast, the least important criterion varied by goal. Health care processes that advanced care that is equitable were rated as least important for reduced mortality (mean [SD], 2.8 [3.2]), improved service effectiveness was least important for increasing function (mean [SD], 3.0 [2.5]), and reflecting long-term health goals was least important for symptom relief (mean [SD],2.0 [2.2]). The top 4 criteria per goal (allowing overlap) were selected with health system advisory group input as the final 7 criteria in round 2 to evaluate attributes of health care processes important for contributing to patient-centered goal domains. In uncertainty simulations, criteria weighted as top 4 or higher within each goal would have been retained in round 2 in some combination between 68% and 81% of repeated iterations for mortality, 75% and 98% for function, and 28% and 97% for symptom relief (eTable 2 in the Supplement).

Respondents proposed 11 additional free-text criteria. These were ineligible for survey 2 based on MCDA criteria principles (8 proposed criteria [73%]) or because they had lower scores than the initial top 4 options (3 proposed criteria [27%]). Therefore, none were added to round 2.

### Prioritizing Health Care Processes According to Patient-Centered Goal Domains

After scoring by the round 1 final criteria, the 53 health care processes varied in final recommended priority ranking by patient-centered goal domain (Table 3; eTables 3-5 in the Supplement). Across all goals, tobacco use screening was the recommended as highest-priority process to complete (mortality: total, 7.4 [27% of iterations as the No. 1 priority in uncertainty simulations, 39% as rank No. 1 or 2]; function: total, 6.9 [49% of iterations as the No. 1 priority, 65% as rank No. 1 or 2]; symptom relief: total, 7.1 [44% of iterations as the No. 1 priority, 61% as rank No. 1 or 2]). In contrast, for all goals, military toxic exposure screening was the lowest-priority process (mortality: total, 3.6 [62% of iterations as No. 53 priority, 88% as rank No. 52 or 53]; function: total, 3.6 [70% of iterations as rank No. 53, 95% as rank No. 52 or 53]; symptom relief: total, 3.8 [63% of iterations as rank No. 53, 90% as rank No. 52 or 53]). Total scores and rankings for the other processes varied by goal.

## Discussion

This consensus statement is the first we know of with recommendations offering a starting position for clinicians to reconcile these perspectives or for systems attempting a more patient-centered framework for their health care processes. Novel findings include variation in what criteria should be important in decision-making around health care processes, relative to 3 broad domains of patient goals. Organizations operationalize health care processes, such as screenings or preventive care, to reflect system priorities around population health or to support performance reporting. Personalizing care for patients with multimorbidity can create tension for clinicians trying to accommodate system or population-health priorities with goals of individual patients.^10,11^ As many health systems and clinicians default to severity, prognosis, or utilization as principle criteria for decision-making,^8,9^ a wider set of criteria differing by these patient-centered goal domains presents an alternative framework. We also developed a highly novel prioritization ranking, linking routine health care processes and patient-centered goal domains. Despite little variation between processes across goal domains, priority ranking substantially varied within each goal and could offer a useful patient-centered approach for triaging among numerous routine processes.^5,7^

Within the past 5 years, national organizational and federal payment structures have increasingly supported care delivery aligned with patient goals, personalized care plans, and patient-centered outcome measures.^49–52^ Our findings have applications for system-level decision-makers and clinicians. System-level decision-makers could use the top overall or a goal-aligned subset of criteria as a patient-centered framework to prioritize existing health care processes or to develop new processes better aligned with patient goals. System-level decision-makers could reconsider seemingly important but ultimately low-priority ranked health care processes, for example, perceived low-rated actionability of sexual trauma screening could result in efforts to clarify referral workflows for positive screenings. Clinicians could apply the priority rankings of health care processes for more efficient patient-centered decision-making prioritizing among, or gaining buy-in for, specific processes if an individual patient’s preferred health goal were known—particularly as health goal elicitation becomes increasingly operationalized.^53–55^ For example, for patients with multimorbidity focused on symptom relief goals, clinicians might decide to spend time on screening for tobacco use and reviewing cessation before addressing health care processes with longer time horizons (eg, glycemic control for diabetes). These efforts, particularly if they are catalyzing conversations, can contribute in aggregate to patient-reported measures of goal alignment,^52^ provide a starting place for clinicians to further individualize decisions around prioritizing processes, or contribute to larger organizational alignment with policies supporting patient-centered care.

This work complements evolving literature attempting to improve or guide primary care capacity for completing routine health care processes for patients with multimorbidity, from a conceptual premise that resources or time will necessitate triaging completion of processes otherwise declared, by definition, of organizational value. At the system level, other VHA primary care studies have explored solutions to extend health care process delivery capacity through technology (eg, automating screenings for electronic delivery),^56^ workflow modification (eg, mailing colorectal cancer home testing kits),^57^ or population health activities (eg, automating mammography screening by default opt-in).^58^ Other efforts have focused on broader, systemic approaches reducing ineffective or inapplicable health care processes.^14,59,60^ At the patient level, there are also increased attempts to personalize decisions around health care processes for patients with multimorbidity (eg, by orienting care around patient-identified goals, priorities, or values, or to end some processes altogether through shared decision-making).^61–63^ Our findings can help advance linkages between individual patient goals and preferences with system-level guidance or demands on care, supporting patients, frontline clinicians, and primary care leaders and policymakers.

Beyond the direct applications of our findings, our methods and approach may be of value to researchers or operational leaders tackling similar complex health care problems and seeking efficient participatory or consensus methods beyond traditional working groups or panels to accommodate conflicting viewpoints, diverse evidence, or disparate inputs. Several aspects of our use of MCDA in this consensus method are notable. While MCDA is established in health-related decision-making,^25,64^ this is a novel application to these topics (patient-centered care, prioritization of tasks). Second, we successfully demonstrated the feasibility of a survey-based consensus-seeking approach rather than more effortful but perhaps less restrictive options, such as facilitated discussions. While we conducted more complex aspects of this study, such as uncertainty simulation, for improved interpretability and rigor of the findings, the basic components of the MCDA approach (selection of health care processes, criteria) could be tailored and replicated with relevant stakeholders and lower-effort methods (eg, emailed worksheets, self-reported grading of uncertainty levels).^25^

### Limitations

This report has some limitations. We acknowledge that our findings were based on a process with a defined scope, incorporating viewpoints from purposely selected participants, and the survey tasks focused on patients with multimorbidity with increased risk of adverse events. SMEs were oriented using common but specific conditions and patient goal domains. We attempted to improve representation of contributors across geography, area of expertise, and perspectives with purposeful recruitment of SMEs and codesign methods via patient and health system advisory groups. However, the goal domains, scope, and participant selection may have affected perspectives on criteria and rankings and limit generalizability or transferability to some patients based on individual health outcome goals, comorbidity, or other factors. Next, we acknowledge some residual uncertainty may result from this technique.^47,48^ We attempted to address critical forms, including applying Monte Carlo simulation with evidence-based uncertainty parameters to assess contributions from differing inputs, and using iterative review with diverse stakeholders. Furthermore, we recognize the VHA-centric perspective for some aspects, such as prioritization of VHA-based guidelines and the team-based care model. While many VHA quality metrics and health care processes are analogous to other systems,^65,66^ this may also affect transferability depending on local context or resources. Despite these limitations, we believe this study provides groundwork for further research or approaches reconciling these seemingly disparate topics.

## Conclusions

This consensus statement describes recommendations from a method reconciling the individual patient-centered perspective with a systematic approach to prioritizing among routine health care processes for patients with multimorbidity. These findings may help clinicians or systems inform decision-making, and our novel approach may be of utility. Future studies could adapt and apply our methods to decision-making around other health goals of importance, incorporate other stakeholder perspectives, or be used to review alternative health care processes.

## Supplementary Material

Supplementary Material **eMethods**. Detailed description of methods
**eTable 1**. Evidence Summary with source citations (provided to subject matter experts in survey 2)
**eTable 2**. Potential criteria and uncertainty simulations around rankings
**eTable 3**. Per-criteria scores and rankings for care processes for mortality goal
**eTable 4**. Per-criteria scores and rankings for care processes for function goal
**eTable 5**. Per-criteria scores and rankings for care processes for symptom relief goal

## Figures and Tables

**Figure.**
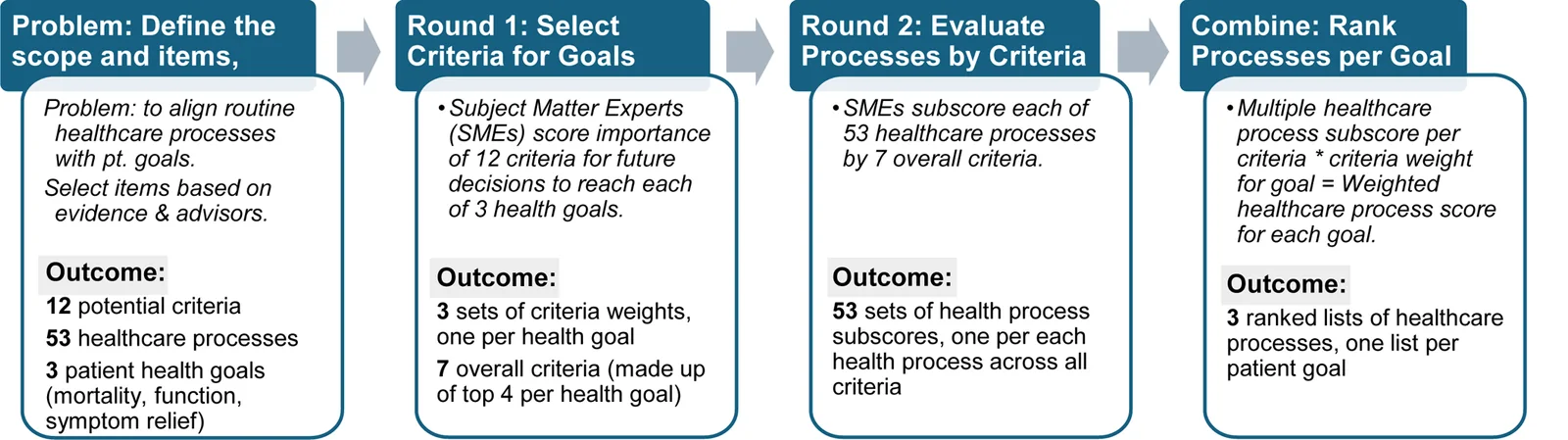
Multi-Criteria Decision Analysis (MCDA) steps

**Table 1.** Subject Matter Expert demographics (N = 20).

Characteristics	n (%)
**Regions**	
West	10 (50)
Midwest	3 (15)
Northeast	5 (25)
South	2 (10)
**Academic institution affiliated**	19 (95)
Professors	8 (40)
Associate or Assistant	11 (55)
**Expertise** ^a^	
Research Investigator	17 (85)
Quality of care, measurement, systems	12 (71)
Multimorbidity, high-risk, geriatrics	12 (71)
Chronic disease management, prevention	8 (47)
Patient-centered or goal-directed care	6 (35)
Clinician	20 (100)
Primary Care / General Internal Medicine	7 (35)
Geriatrics Specialty	9 (45)
Palliative Care Specialty	3 (15)
Psychiatry	1 (5)
Leadership	10 (50)
Departmental or Division Chair / Service Chief	5 (25)
Associate Chief / Director level	5 (25)

a Multiple domains may be selected.

**Table 2.** Importance of criteria to evaluate healthcare processes for contribution to a patient-centered health goal (scale 1–100, higher as more important).

Criteria *(“To contribute towards outcome [at right], it is most important for healthcare processes to…”)*	Mortality Mean (SD)	Function Mean (SD)	Symptoms Mean (SD)	(Included in Survey 2) ^a^
Be directly actionable by clinicians/team	25.3 (37.6)	16.4 (5.5)	15.2 (5.2)	Yes
Reflect high-prevalence health issues (affecting the most patients possible)	11.7 (8.2)	10.0 (6.0)	9.5 (7.9)	Yes
Be highly valid (measuring what they are supposed to measure)	10.2 (7.6)	8.2 (6.7)	13.0 (4.1)	Yes
Be grounded in published, high-quality evidence	8.8 (8.8)	7.4 (6.3)	10.3 (5.1)	Yes
Be highly reliable (provides same results if measured repeatedly)	7.7 (8.2)	4.2 (3.8)	5.0 (4.4)	No
Advance safety and reduce harm to patients	7.0 (7.5)	3.4 (4.7)	13.3 (16.8)	Yes
Reflect longer-term health goals (e.g., outcome measures)	6.7 (10.8)	12.6 (5.0)	2.0 (2.2)	Yes
Contribute to timely, efficient, and accessible care	6.2 (6.2)	9.0 (6.6)	6.5 (5.2)	No
Be easy to complete as measurement tasks and/or measured via EHR data	5.0 (6.3)	11.0 (5.8)	8.8 (3.4)	Yes
Improve service effectiveness (optimize appropriate care, minimize waste/medical burden)	4.5 (6.6)	3.0 (2.5)	7.2 (4.5)	No
Reflect shorter-term goals (e.g., process measures)	4.2 (8.0)	7.6 (6.4)	4.2 (3.9)	No
Advance care that is equitable (accurate and fair across patient subgroups)	2.8 (3.2)	7.4 (5.6)	5.0 (4.4)	No

a Allowing for overlap, top 4 criteria selected per goal used in survey Round 2.

**Table 3.** Priority ranking of ambulatory healthcare processes based on contribution toward specific patient-centered health goal (lower-numbered rank as greater priority).

Care process	Mortality		Function		Symptom Relief	
	Rank	% +/- 1^a^	Rank	% +/- 1^a^	Rank	% +/- 1^a^
Tobacco use screening, evaluation of usage	1	39.4	1	65.1	1	61.3
Influenza immunization in patients ≥ 65y	2	19.9	6	19.0	2	32.3
Pneumococcal immunization	3	22.4	3	21.1	4	23.2
Diabetes: kidney health screening	4	17.5	2	24.2	6	20.0
HIV testing in average-risk patients	5	14.9	4	22.5	7	17.9
Diabetes: glucose control	6	15.6	5	24.4	3	25.6
Hepatitis B immunization in high-risk patients	7	12.5	8	15.0	5	19.3
Depression screening	8	11.9	7	17.1	8	15.6
Diabetes: retinal screening	9	11.0	12	11.6	11	11.7
Influenza immunization in adult patients < 65y	10	10.0	11	13.0	10	14.5
Tobacco cessation intervention	11	12.8	10	15.0	9	15.5
Housing and food insecurity screening	12	10.0	13	11.6	13	12.0
Fall screening, function assessment in older adults	13	10.8	14	11.5	12	12.2
Blood pressure control in hypertensive patients	14	9.8	9	15.8	14	14.4
Diabetes: foot examination	15	8.8	15	10.9	16	-
Cervical cancer screening in eligible patients	16	-	18	-	15	9.7
HPV immunization	17	-	20	-	17	-
Meningococcal immunization in at-risk patients	18	-	17	-	19	-
Blood pressure control in diabetes	19	-	16	-	18	-
Cholesterol monitoring in at-risk patients	20	-	23	-	30	-
Beta blockade for prior myocardial infarction	21	-	19	-	23	-
Breast cancer screening in eligible patients	22	-	21	-	22	-
Tdap immunization	23	-	22	-	21	-
Colorectal cancer screening in average-risk patients	24	-	25	-	20	-
Osteoporosis screening in women >/= 65y	25	-	24	-	24	-
Opioids: narcotic safety	26	-	27	-	25	-
Opioid use disorder: MAT	27	-	28	-	26	-
Hepatitis C screening in average-risk patients	28	-	26	-	31	-
Alcohol misuse/use disorder screening	29	-	29	-	28	-
Follow-up after positive suicide risk screening	30	-	30	-	27	-
Abdominal aortic aneurysm screening, at-risk patients	31	-	32	-	36	-
Chlamydia screening in young women	32	-	35	-	29	-
Lung cancer screening in at-risk patients	33	-	34	-	33	-
Statin therapy for high-risk patients	34	-	31	-	37	-
Medication reconciliation	35	-	38	-	34	-
Intimate partner violence screening	36	-	36	-	32	-
Liver cancer screening in high-risk patients	37	-	37	-	35	-
High-risk patients referred to palliative care	38	-	33	-	39	17.9
Heart failure: weight monitoring education	39	11.9	39	10.6	38	-
Heart failure: ACE or ARB use	40	11.3	41	14.3	41	17.7
Urinary incontinence in older adults	41	13.3	42	15.9	40	17.1
Annual suicide risk screening	42	11.9	40	13.4	42	19.7
Alcohol misuse/use disorder intervention	43	13.0	29	19.6	43	19.4
Follow-up after positive PTSD/Depression screen	44	13.3	45	24.5	45	27.2
PTSD screening	45	15.8	44	23.7	44	21.3
Depression monitoring	46	19.5	46	33.5	46	28.3
Obese patients participating in weight management	47	25.3	48	44.3	49	52.1
Diabetes: glucose monitoring	48	25.8	47	37.1	47	41.7
Hepatitis B immunization in average-risk patients	49	31.3	49	54.4	48	54.1
Traumatic brain injury screening in at-risk patients	50	42.5	50	69.4	50	69.4
Sexual trauma screening	51	52.1	51	69.4	51	77.2
Embedded fragment screening	52	65.9	52	88.3	52	87.1
Military toxic exposures screening	53	88.1	53	95.0	53	89.5

a Describes percent of simulated iterations as within plus- or minus-one of original rank; only top 15 and bottom 15 processes included in uncertainty simulation.

## Key Points

### Question

In busy clinic settings, how can routine health care processes (eg, screenings, chronic disease care) be aligned with patients’ preferred health goals?

### Findings

This consensus statement used a consensus-based method among topic experts and operational and patient advisory groups to identify 7 attributes (actionability, safety impact, completion ease, health issue prevalence, evidence quality, long-term health outcome impact, and validity) recommended to evaluate how 53 processes align with 3 goal domains (mortality, symptoms, function). Processes varied in priority per goal, when reviewed according to those attributes.

### Meaning

These findings suggest that reconciliation of routine health care processes with patient health goals offer a novel patient-centered approach to triaging effort among numerous processes.
