# Supplementary material for: Patient-Centered Prioritization of Health Care Processes for Multimorbidity
Source: JAMA Netw Open. Author manuscript; Available in PMC 2026 Sep 8. (PMC13552484; doi:10.1001/jamanetworkopen.2025.49693)
Supplement: Supplementary file 1 [file NIHMS2195427-supplement-Supplementary_Material.docx]

**Supplementary material**

**eMethods. Detailed description of Methods.**

**eTable 1. Evidence Summary with source citations (provided to Subject Matter Experts in Survey 2).**

**eTable 2. Potential criteria and uncertainty simulations around rankings.**

**eTable 3. Per-criteria scores and rankings for care processes for Mortality goal.**

**eTable 4. Per-criteria scores and rankings for care processes for Function goal.**

**eTable 5. Per-criteria scores and rankings for care processes for Symptom Relief goal.**

**eMethods. Detailed description of Methods.**

For brevity, an overview of methods for the consensus approach are provided in the main manuscript, with expanded, detailed methodology outlined here. References appearing here are included at the end of the supplementary material.

*Participants:*

For Multi-Criterion Decision Analysis, three groups provided perspectives: Subject Matter Experts (SMEs); a patient advisory group; and a health systems advisory board. SMEs were purposefully recruited based on national reputations, determined by publications on public search engines, academic profiles, and/or health system leadership positions. Inclusion criteria included at least one of: a minimum of 10 years of expertise as a nurse or physician in a specialty related to multimorbidity and outpatient care (e.g., internal medicine, geriatrics); Associate academic rank or higher as a research investigator with national reputation in a related area (e.g., chronic disease management, patient-centered care); or a supervisory health leadership role (e.g., Chief of Primary Care). Recruitment was stratified across categories (clinical, research, leadership), though many fulfilled multiple roles. Eligibility criteria were reviewed with, and nominations for candidates were also solicited from health system advisory partners. Eligible individuals were evaluated and final candidates selected by the study team (LS, MT, KN, AMR); enrollment targeted 15-21 SMEs based on prior literature.^1,2^ After determining eligibility, an invitation email was extended by the study lead, with one follow-up for non-response. Interested individuals replied over email to confirm eligibility and provide consent. Recruitment emails were sent to 42 SMEs, 21 (50%) enrolled. Of these, one participant did not respond in either round and was removed. After enrolling, participants were sent two rounds of an electronic, English-language Qualtrics survey (content below). Round 1 was completed between February 26-April 2, 2024; Round 2 between June 3-July 26, 2024. SME identities were anonymized for publication as a condition of regulating bargaining units. Participants were blinded to the identity of other SMEs. In Round 1, 18/21 (86%) SMEs participated; in Round 2, 19/21 (90%) SMEs participated; overall, 20/21 (95%) SMEs participated in at least one round. Participating SMEs were offered an optional $45 for completing both rounds, contingent on eligibility for compensation.

Patient advisory input was solicited through two sessions (2021, 2023) with the VA Puget Sound Veteran Engagement Board (VEG). The VEG consists of 6-10 diverse Veterans representing the VHA patient community including individuals with multimorbidity, trained as research consultants and compensated by the institution. During facilitated, online group discussion sessions the VEG provided input on a) patient-centered health goal domains, and b) decision criteria.

A health system advisory board was also formed for the parent research study that supported this consensus method, comprised of national and local clinical operational leaders and organizational representatives (co-authors: LC, SH, TS, TS, and KR). This board met virtually as a group every 6 months for feedback, including on criteria and healthcare process selection, with additional asynchronous individual ad hoc input on materials and design throughout. Piloting of the survey content was completed with two health system advisors for clarity; responses were not included in consensus. Feedback was obtained by facilitated discussion, anonymous voting, and/or qualitative (written) comments.

*Multi-criterion decision analysis*

We began by selecting potential criteria and healthcare processes. We developed a list of 42 potential criteria based on literature.^3–11^ After consolidation for duplicates, 28 possible criteria were discussed with patient and health system advisors. Twelve criteria were selected, aligned with MCDA best-practices (3-19 criteria).^12^ We included all healthcare processes (n=373) used nationally in 2023 in the U.S. Veterans Health Administration (VHA), including performance metrics, population-health measures, or clinical reminders - electronic health record embedded tasks for eligible patients operationalizing clinical practice guidelines or addressing VHA priorities such as mandated screenings.^13^ Two study members separately reviewed each process denominator with the following inclusion criteria: applicability to populations with multimorbidity, actionability by individual patients and clinicians, non-composite (not multiples of many domains), and outpatient care use. Of these, 319 were categorized similarly by both reviewers (kappa=0.72).^14^ Disagreements were resolved by discussion. Overall, 91 specific healthcare processes met all inclusion criteria. These were categorized by disease- or preventive category (e.g., diabetes), and combined if similar, to create more universal constructs (e.g., one healthcare process for ‘glycemic control in patients with diabetes’, rather than two separate processes describing different HgbA1C targets of < 8% and < 9%). After feedback and iterative input from patient and health system advisory boards, 53 healthcare processes were included in MCDA.

Study objective one, operationalized into Survey 1, encompassed selecting and weighting the importance of decisional criteria per health goal domain. Each SME received one of three possible survey versions, differing by including one of three patient-centered health goal domains: mortality (extending life); improving or maintaining physical and cognitive functioning; and symptom reduction including pain and mental-health symptoms. Health goal domains were selected based on literature^15–19^ and patient advisory group input. All survey versions then asked SMEs to weigh the importance of the 12 criteria by allocating up to 100 points (more points for greater import), if aligning care to a specific health goal domain. SMEs were asked to consider all patients with multimorbidity (≥ 2 chronic diseases) in responses. However, if helpful, they were asked to consider hypothetical Veterans with multimorbidity as ≥ 2 of diabetes, hypertension, chronic kidney disease (CKD), and/or depression, and ≥ 75% risk of mortality or hospitalization in the next three months using a validated VHA risk-prediction score.^20^ This combination was selected as representing common, prevalent diseases clustering among high-risk Veterans,^21^ likely to have interactions in disease treatment plans^22^ (and therefore more benefit from focusing on patient-centered health goals). SMEs could also free text in alternative criteria and assign additional points, if desired. We normalized responses to a 100-point scale and calculated mean and standard deviations per criteria, for each goal. Free-text responses were reviewed by two study members (LS, MT) according to principles governing creation of criteria for MCDA: complete, non-redundant, non-overlapping, and assessable independently.^23^ We reviewed results with the health system advisory board, and to limit survey burden, selected the top 4 criteria per health goal domain. With overlap between multiple “top 4” criteria between the outcomes, this led to a final 7 criteria for Survey 2.

Study objective two, or Survey 2, intended to prioritize healthcare processes by scoring them according to criteria from Survey 1. As in Survey 1, SMEs were asked to consider patients with multimorbidity in general, with the optional more detailed hypothetical definition (≥ 2 diabetes, hypertension, kidney disease, or depression plus ≥ 75% adverse risk). We included the 53 healthcare process constructs for scoring by the 7 final criteria from Survey 1. For Survey 2, SMEs were provided an evidence summary for the healthcare processes (described below). Healthcare processes were divided across SMEs to reduce survey burden, each SME received a randomized selection of up to 11 healthcare processes. SMEs scored each healthcare process on a 10-point Likert scale from worst- to best-performing per criterion. To reduce response-order effect, presentation order of healthcare processes and criteria was randomized.

After completion of Survey 1 (criteria and weights) and Survey 2 (scores), we combined results. The mean criteria importance weight per health goal domain (Mortality, Function, or Symptom Relief) was multiplied by the healthcare process score for that criteria.^12^ These values were then summed for each healthcare process, with a higher total sum (maximum of 10.0) representing better alignment with a patient-centered health goal domain. This sum was translated to a priority ranking of healthcare processes, varying in order by each of the three health goal domains.

To determine the impact of potential uncertainty in weighting and healthcare process scoring within MCDA,^24^ we incorporated Monte Carlo simulation modeling for both the criteria weights and the rank (total sums) of the healthcare processes.^25^ We specified the mean and standard deviation of the observed importance weight for each criterion, or the observed total weighted sum for each healthcare process after scoring by criteria, to generate random values from the inverse of the normal cumulative distribution (5,000 iterations). We then calculated the number of random iterations and the proportion of iterations equal to the original rank. We present uncertainty as the proportion of total iterations that resulted in plus one position or minus one position from the original rank. Higher percentages reflect lower uncertainty in the weighting or scoring outcome.

*Evidence summary*

To assist SMEs, we developed an evidence summary for the healthcare processes. We searched disease-based and preventive care clinical practice guidelines related to the healthcare processes using related categories as keywords (e.g., diabetes, colorectal cancer) using major search engines for peer-reviewed and grey literature (PubMed, Google Scholar) for the most recent published guidelines through January 19, 2023 in the following priority order: VHA-based guidelines, non-VHA national professional bodies (U.S.), then international professional bodies. Twenty clinical practice guidelines were reviewed by three study members for statements and evidence supporting each healthcare process, and relevant excerpts extracted and organized by priority order (VHA, non-VHA U.S., non-U.S.). These findings were summarized into an evidence matrix provided during survey Round 2 to participants (described above). The matrix included a description of the healthcare process, target population of interest and disease prevalence based on the official VHA clinical documentation, and a summary of the evidence from the clinical practice guidelines and associated strength of evidence grade. The evidence matrix with guidelines and citations is included in eTable 1.

**eTable 1. Evidence Summary with source citations (provided to Subject Matter Experts in Survey 2).**

| **Healthcare Process** | **Description** | **Evidence discussion** | **Level of Evidence & Guideline Source** | **Prevalence (per 1,000)** | **Target Pop.** | **Source** |
| --- | --- | --- | --- | --- | --- | --- |
| **Abdominal aortic aneurysm screening in at-risk patients** | Screening for abdominal aortic aneurysm in men between 65-75 years, who are current or former smokers. | Abdominal aortic aneurysm (AAA) rupture predicts 18%-32% mortality. However, the prevalence of AAA disease has been decreasing, largely due to cardiovascular risk factor management and lower smoking rates. | USPSTF: "B" (moderate benefit). | 2.2 | Male US Veterans (age-specific) | ^26–28^ |
| **Alcohol misuse/use disorder intervention** | Intervention (counseling and/or medication) for alcohol misuse/use disorder | Brief alcohol counseling (reviewing related risks and advice to abstain or limit consumption) has been shown to decrease drinking and adverse health outcomes. Brief alcohol counseling was identified by the CDC and AHRQ as a top 10 national prevention priorities based on clinically preventable burden of disease and cost-effectiveness. Evidence-based pharmacotherapies, such as naltrexone or disulfiram for alcohol use disorder improve clinical outcomes. | VA/DOD: "Strong for" (high confidence). | 150 | All US Veterans | ^29,30^ |
| **Alcohol misuse/use disorder screening** | Annual screening for alcohol misuse/use disorder (AUDIT-C use) among all outpatients | Around 15% of VA primary care patients screen positive for alcohol misuse, which is associated with increased mortality, psychosocial, legal, or employment problems, personal suffering, and adverse health outcomes like cirrhosis, pancreatitis, neuropathy, and cardiomyopathy. Brief counseling after screening decreases drinking and improves health outcomes. Many drinkers who are not alcohol dependent have medical or social problems attributable to alcohol. | Alcohol use screening (general) - USPSTF: "B" (moderate benefit). AUDIT-C screener (specifically) - VA/DOD: "Strong for" (high confidence). | 150 | All US Veterans | ^29,31,32^ |
| **Annual suicide risk screening** | Annual suicide risk screening in all outpatients, including completion of a Columbia Suicide Severity Rating Scale (C-SSRS) Annual Screen or comprehensive suicide risk evaluation (CSRE) | Suicide in Veterans exceeds the general population rate (21% higher, both in males and females). Use of the specific screening tool used in the VA, the Columbia screen, is not specifically mentioned in the VA/DOD Clinical Practice Guidelines (2019). | Validated screening tool (general) - VA/DOD: "Weak for" (suggested conditionally). Columbia screener (specifically) - VA/DOD: No recommendation. | 0.27 | All US Veterans | ^33,34^ |

| **Healthcare Process** | **Description** | **Evidence discussion** | **Level of Evidence & Guideline Source** | **Prevalence (per 1,000)** | **Target Pop.** | **Source** |
| --- | --- | --- | --- | --- | --- | --- |
| **Beta blockade for prior myocardial infarction** | Patients with outpatient beta-blockade prescription for 6+ months after discharge for an acute myocardial infarction | Beta blockers (BB) reduce infarct size, early mortality (sudden cardiac death risk reduces 47 to 30%), and also reduce the risk of death when continued long term. Continuing BB during and after hospitalization for all patients with ST-Elevation myocardial infarction (STEMI) and with no contraindications to use is a "B" level of evidence (moderate quality) from the American Heart Association (2013). For patients with Non-STEMI/Acute Coronary Syndrome, continuing BB is recommended in patients with normal left ventricular function ("C" level of evidence (limited data/expert consensus), AHA, 2014). | For prior STEMI - Am. Heart Assoc.: "B" (moderate quality).  For prior non-STEMI - Am. Heart Assoc.: "C" (limited data/expert consensus) | 71 | All US Veterans (select diagnoses) | ^31,35–37^ |
| **Blood pressure control in diabetes** | Patients with diabetes with most recent measured blood pressure < 140/90 mmHg | To reduce risk of stroke, cardiovascular events, and mortality for individuals with diabetes and hypertension at lower risk for cardiovascular disease (CVD, 10-year ASCVD risk < 15%), it is recommended to treat to at minimum a blood pressure target of < 140/90 mmHg. | Am. Diabetes Assoc.: "A" (high-quality). | 205 | All US Veterans (select diagnoses) | ^38,39^ |
| **Blood pressure control in hypertensive patients** | Control of blood pressure in all eligible outpatients patients with elevated blood pressure (>/= 140/90 mmHg) at most recent visit | About 1 in 3 U.S. adults have hypertension. Controlling hypertension reduces morbidity, such as reduction of heart attacks, stroke and kidney disease, as well as cardiovascular mortality (for males and females by 30.4% and 38.0%, respectively). Tighter control (< 130/80) is recommended in patients with diabetes or cardiovascular disease risk factors across multiple Clinical Practice Guidelines. | Am. Heart Assoc.: "B" (moderate quality). | 431 | All US Veterans | ^35,39^ |
| **Breast cancer screening in eligible patients** | Women screened for breast cancer (eligible women 45-54y with annual screening, or 55-74y with biennial screening or discussion of annual screening option) | Breast cancer is the second most common type of cancer among American women. Advancing age is the primary risk factor for breast cancer, which is most commonly diagnosed between 55-64y. Mammograms are the best method to detect early breast cancer, which can provide women with a greater range of treatment options and reduces the risk of dying from breast cancer by 20%. | For ages 40-49y - USPSTF: "C" (neither for nor against). For ages 45y+ - Am. Can. Society: "Qualified".  For ages 50y+ - USPSTF: "B" (moderate benefit). | 120 | Women US Veterans (age-specific) | ^40,41^ |

| **Healthcare Process** | **Description** | **Evidence discussion** | **Level of Evidence & Guideline Source** | **Prevalence (per 1,000)** | **Target Pop.** | **Source** |
| --- | --- | --- | --- | --- | --- | --- |
| **Cervical cancer screening in eligible patients** | Cervical cancer screening at appropriate intervals in eligible women patients age 21-64y (includes HPV testing for age 30 and greater) | Cervical cancer is the fourth most common type of cancer in females worldwide, and around 14,000 cases occur in the US annually. Cervical cancer screening is associated with higher cure rates for invasive disease and lower mortality. HPV testing is very sensitive, and improves detection of cervical intraepithelial neoplasia compared to Pap testing alone. | USPSTF: "A" (substantial benefit). | 0.04 | Women US Veterans (age-specific) | ^42^ |
| **Chlamydia screening in young women** | Chlamydia screening in eligible women patients ages 18-24y | The USPSTF recommends chlamydia screening in sexually active females aged 24y or younger and in older women at increased risk for infection. | USPSTF: "B" (moderate benefit). | 19.5 | Women US Veterans (age-specific) | ^43,44^ |
| **Cholesterol monitoring in at-risk patients** | Cholesterol panel testing in patients with cardiovascular disease or diabetes. | In patients with cardiovascular disease, lipid profiles vary as much as 20% with repeat measurement, and the data behind optimal frequency of lipid testing is not clear. Too-frequent lipid testing can increase patient anxiety and contribute to healthcare waste, given relatively minor effects of lipids on overall cardiovascular risk scores. In patients with diabetes, lipid testing for screening and monitoring for lipid-lowering medication use is recommended with an "E" (expert opinion) by the American Diabetes Association. Lipid testing is recommended as "E" (expert opinion) for patients with heart failure by the American College of Cardiology. Routine monitoring lipids for patients on statin is a "weak against" recommendation in VA/DOD guidelines. | In diabetes - Am. Diabetes Assoc.: "E" (expert opinion).  In heart failure - Am. College Cardiology: "E" (expert opinion).  In all patients on statins - VA/DOD: "Weak against" | 92 | All US Veterans (select diagnoses) | ^31,35,45–47^ |
| **Colorectal cancer screening in average-risk patients** | Colorectal cancer screening in eligible average-risk patients, ages 45-74y | Colorectal cancer accounts for 8% of all cancer deaths in the US, and lifetime incidence in patients at average risk is approximately 4%. Age is a major risk factor, and incidence begins to increases significantly between 40 to 50. Screening at 45y is recommended by the American Cancer Society and the USPSTF. | USPSTF: "A" (substantial benefit). | 0.42 | All US Veterans (age-specific) | ^48^ |

| **Healthcare Process** | **Description** | **Evidence discussion** | **Level of Evidence & Guideline Source** | **Prevalence (per 1,000)** | **Target Pop.** | **Source** |
| --- | --- | --- | --- | --- | --- | --- |
| **Depression screening** | Screened annually for depression (PHQ-2) in all outpatients | Major depression is a highly prevalent, morbid, and costly illness that is often unrecognized and inadequately treated; up to 50% of cases can go unrecognized. Depressed medical patients have increased disability, health-care utilization, and mortality from suicide and other causes, as well as reduced productivity and health-related quality of life. Depression screening may improve recognition and hasten treatment. | VA/DOD: "Strong for" (high confidence) | 123 | All US Veterans | ^49,50^ |
| **Diabetes: foot examination** | Patients with diabetes with foot health evaluation and examination (monofilament, pulses, inspection) | The lifetime risk of a foot ulcer for patients with type 1 or 2 diabetes may be up to 34%. In patients with diabetes, foot ulceration increases mortality risk 2.5-fold, and is associated with lower physical and mental health-related quality of life scores compared to those without foot disease. | American Diabetes Association: "B" (moderate quality).  VA/DOD: "Strong for" (high confidence). | 70 | All US Veterans (select diagnoses) | ^38,51–53^ |
| **Diabetes: glucose control** | Patients with diabetes with controlled blood glucose (most recent HbA1c < 9%) | Every 1% drop in glycated hemoglobin (HbA1c) is associated with improved microvascular and macrovascular outcomes; targeting HbA1c </= 7% reduces these outcomes for most adults. Less stringent targets (e.g., HbA1c 7.0-8.5%) may be appropriate for individuals with established complications, comorbidity, or limited life expectancy, are recommendations supported by the VA/DOD Clinical Practice Guidelines and American Diabetes Association. Overtreatment of diabetes is associated with increased mortality and harm to patients by falls, injury, or contributing to dementia risk. | Target A1C 7.0-8.5% for at-risk patients - VA/DOD: "Strong for" (high confidence). Target A1C 7.0-8.5% for at-risk patients - Am. Diabetes Assoc.: "B" (moderate quality). | 205 | All US Veterans (select diagnoses) | ^38,52,54^ |
| **Diabetes: glucose monitoring** | Annual HgbA1C monitoring in patients with diabetes | The A1C has a strong predictive value for diabetic complications, and is reflective of long-term (120d) average glycemia, though does not reflect variability or hypoglycemia well, which is associated with increased mortality and harm to patients by falls, injury, or contributing to dementia risk. Twice yearly testing is recommended by the American Diabetes Association for stable adults with diabetes. More frequent testing (3mo) is recommended for unstable glycemic or uncontrolled patients. | Testing every 6 mo, stable patients - Am. Diabetes Assoc.: "E" (expert opinion).  Testing every 3 mo., unstable patients - Am. Diabetes Assoc.: "E" (expert opinion). | 205 | All US Veterans (select diagnoses) | ^38,54,55^ |
| **Healthcare Process** | **Description** | **Evidence discussion** | **Level of Evidence & Guideline Source** | **Prevalence (per 1,000)** | **Target Pop.** | **Source** |
| **Diabetes: kidney health screening** | Patients with diabetes ages 18-85y with annual kidney health evaluation (e.g. microalbuminuria testing) | Approximately 30% of patients with type II diabetes have CKD. Monitoring for urine albumin and/or GFR can be helpful in diagnosis, as the course can be variable and abnormalities in the GFR or protein excretion can occur independently, progress, or even regress over the course of nephropathy. However, meeting the related quality metric in observational studies had no association with reduced mortality among a Medicare population. Annual screening (or twice annually for patients with abnormal function) is a "B" (e.g., well-conducted cohort study) to "C" (e.g., uncontrolled studies) level of evidence from the American Diabetes Association. | Am. Diabetes Assoc.: "C" to "B" level (moderate quality). | 62 | All US Veterans (select diagnoses) | ^38,56,57^ |
| **Diabetes: retinal screening** | Patients with diabetes ages 18-75y with recommended retinal screening by appropriate provider (e.g., dilated exam by eye care professional) | Diabetic retinopathy is a leading cause of impaired vision in patients 25-74 years; approximately 60% of patients with diabetes have one or more of the ocular complications associated with the disease. In older adults, higher levels of meeting the quality metric for diabetic eye screening was significantly associated with 6% lower adjusted odds of mortality. Every-other year screening for retinopathy is recommended among patients who have had no retinopathy on all previous examinations, or more frequent examinations in patients with risk factors for increased rate of progression of retinopathy. Patients with existing retinopathy should be examined at intervals according to the level of retinopathy. | Am. Diabetes Assoc.: "B" (moderate quality). | 123 | All US Veterans (select diagnoses) | ^38,57–59^ |
| **Embedded fragment screening** | Screening for military service-related embedded fragments from blast or bullet injuries in all outpatients | Service members may have embedded fragment wounds as the result of small arms fire or a blast or explosion caused by an IED, rocket propelled grenade (RPG), landmine, or grenade. Retained fragments can cause morbidity and are also associated chronic elevated blood lead levels. In particular, service members who served in Iraq and Afghanistan are at increased risk. | No clinical practice guidelines. | 13 | All US Veterans | ^60^ |

| **Healthcare Process** | **Description** | **Evidence discussion** | **Level of Evidence & Guideline Source** | **Prevalence (per 1,000)** | **Target Pop.** | **Source** |
| --- | --- | --- | --- | --- | --- | --- |
| **Follow-up after positive PTSD/Depression screen** | Follow-up assessment by a provider, after a positive PTSD/depression screen | Major depression is a highly prevalent, morbid, and costly illness that is often unrecognized and inadequately treated; up to 50% of cases can go unrecognized. Depressed medical patients have increased disability, health-care utilization, and mortality from suicide and other causes, as well as reduced productivity and health-related quality of life. Depression screening may improve recognition and hasten treatment. PTSD in US Veterans is commonly associated with combat history, and rates of PTSD in Veterans exceed the US population. Early intervention is intended to prevent chronic PTSD and its complicating disorders and functional problems, including major depression, suicide, substance use disorder, family violence, and homelessness. | VA/DOD: "Weak for" (suggested conditionally). | 59 | All US Veterans | ^50,61,62^ |
| **Follow-up after positive suicide risk screening** | Timely follow-up assessment and safety planning after positive suicide risk screening or in high-risk patients | Suicide in Veterans exceeds the general population rate (21% higher, both in males and females). Comprehensive risk factor assessment as part of suicide evaluation is a "strong" recommendation (high confidence) in the VA/DOD Clinical Practice Guidelines, though there is "insufficient" evidence on the use of risk stratification as part of determining the level of suicide risk. Crisis response plans are "weakly" recommended (suggested, conditionally) by the VA/DOD Clinical Practice Guidelines for suicide. | Risk factor assessment - VA/DOD: "Strong for" (high confidence).  Crisis response plans - VA/DOD: "Weak for" (suggested conditionally). | 0.27 | All US Veterans | ^34,63^ |
| **Heart failure: ACE or ARB use** | Patients with reduced left ventricular ejection fraction (< 40%) on angiotensin converting enzyme inhibitor (ACE inhibitor) or an angiotensin II receptor antagonist (ARB or AIIRA) at most recent outpatient visit, if without contraindications | ACE inhibitors are recommended in patients with heart failure with reduced ejection fraction (HFrEF), unless contraindicated, to reduce morbidity, symptom burden, and mortality. ARBs are appropriate in patients intolerant of ACE inhibitors. In patients with reduced LVEF, reductions in mortality have ranged from 19-30% between 2-4 years later, compared to placebo. | Am. College Cardiology / Am. Heart Assoc.: "A" (high quality). | 50 | All US Veterans (select diagnoses) | ^31,64–66^ |

| **Healthcare Process** | **Description** | **Evidence discussion** | **Level of Evidence & Guideline Source** | **Prevalence (per 1,000)** | **Target Pop.** | **Source** |
| --- | --- | --- | --- | --- | --- | --- |
| **Heart failure: weight monitoring education** | Patients with congestive heart failure educated on obtaining daily weights at home | Overall, education of patients with heart failure may result in lower hospitalization, but has not been shown to impact mortality. Longer term strategies are more impactful. Self-monitoring with daily weight measurement for heart failure is aimed at early intervention to avoid hospitalizations. Weight gain at home (particularly > 10 lb. in a week) has been associated with increased odds of hospitalization, although other studies have shown less of an effect. Few heart failure patients outside of trials are able to maintain daily weight measurement, in practice, with real-world observational studies reporting rates of around 14% adherence to daily weights. | Am. Heart Assoc.: "B" (moderate quality). | 50 | All US Veterans (select diagnoses) | ^64,65,67^ |
| **Hepatitis B immunization in average-risk patients** | Hepatitis B blood serology or immunization if not already immune, in average-risk patients ages 19-59y | Universal Hepatitis B Vaccination is recommended in adults aged 19-59 years routinely as of March 2022 in response to higher incidence rates in adults without recognizable risk factors. Serologic carrier rates in the US are < 2% overall, and rates in Veterans exceed the US population. | Advisory Committee on Immunization Practices (ACIP): Recommended. | 10 | All US Veterans (age-specific) | ^68,69^ |
| **Hepatitis B immunization in high-risk patients** | Hepatitis B immunization (if not already immune), in high-risk patients (advanced chronic kidney disease, HIV, or Hepatitis C) | Hepatitis B Vaccination is recommended for high-risk adults. Those with Hepatitis C are at increased risk for HBV reactivation if treated with antiviral therapy, and more severe liver disease if co-infected. Adults with HIV are considered high-risk to acquire Hepatitis B, which is spread primarily through sexual intercourse and injection drug use in low-prevalence areas. People undergoing dialysis or predialysis have increased percutaneous or mucosal risk for exposure through blood-borne transmission. Serologic carrier rates in the US are < 2% overall, and rates in Veterans exceed the US population. | Advisory Committee on Immunization Practices (ACIP): Recommended. | 10 | All US Veterans (age-, diagnosis-specific) | ^68,69^ |
| **Hepatitis C screening in average-risk patients** | Hepatitis C virus screening for all eligible patients ages 18-79y | HCV is three times more prevalent among Veterans in VA care, with a prevalence of over 5%, than in the general US population. Universal screening is recommended by the CDC. The USPSTF recommends screening universally until age 79y, then amongst those with increased risk factors after age 80y (e.g., injection drug use). | USPSTF: "B" (moderate benefit). | 54 | All US Veterans (age-specific) | ^70–72^ |
| **Healthcare Process** | **Description** | **Evidence discussion** | **Level of Evidence & Guideline Source** | **Prevalence (per 1,000)** | **Target Pop.** | **Source** |
| **High-risk patients referred to palliative care** | Patients with high predicted risk (98% percentile or higher in 90 days) of adverse outcomes or mortality referred to palliative care or hospice | High-quality evidence supports the impact of home-based palliative care on home death; referrals facilitate access to services for growing numbers of frail elderly. This measure is recommended in the VA as a population health based assessment for providers to consider if a patient needs palliative care. | No clinical practice guidelines. | 81 | All US Veterans (select diagnoses) | ^73^ |
| **HIV testing in average-risk patients** | HIV testing for all patients at least once in a lifetime | As many as 15% of HIV-infected persons in the US are unaware of their HIV infection. Within the US, nearly 60,000 veterans have an HIV infection. A one-time screening is recommended for patients without identifiable risk factors for patients between 15-65y. | USPSTF: "A" (substantial benefit). | 10 | All US Veterans (age-specific) | ^74,75^ |
| **Housing and food insecurity screening** | Screening for insecurity or access to housing and food in all outpatients | In 2019, 2.4% of veterans engaged in VA care were experiencing homelessness. In 2008, 24% of Veterans reported food insecurity. Higher rates of food insecurity have been reported among Veterans of Iraq and Afghanistan (27%), women Veterans (28%), homeless and formerly homeless Veterans (49%) and Veterans with serious mental illness (35%). | No clinical practice guidelines. | 240 | All US Veterans | ^76,77^ |
| **HPV immunization** | Human papillomavirus immunization (HPV) in all eligible patients without contraindications | In 2014, prevalence of high-risk genital HPV for adults aged 18–59 was 25.1% in men and 20.4% in women, respectively. Optimal vaccination timing is before sexual debut; Evidence for vaccination in adults > 26y is strongest for those with few prior sexual risk factors or risk for obtaining repeat exposure to HPV (such as those with few, low-risk sexual contacts, or health care providers with recurrent HPV occupational exposures). | Advisory Committee on Immunization Practices (ACIP): Recommended. | 245 | All US Veterans | ^78,79^ |
| **Influenza immunization in adult patients < 65y** | Influenza immunization annually in all eligible patients, 19-64y | Annual influenza vaccination is associated with reduced influenza mortality and morbidity, as well as reduced risk of adverse cardiovascular events during flu season. | Advisory Committee on Immunization Practices (ACIP): Recommended. | 5 | All US Veterans (age-specific) | ^80–82^ |

| **Healthcare Process** | **Description** | **Evidence discussion** | **Level of Evidence & Guideline Source** | **Prevalence (per 1,000)** | **Target Pop.** | **Source** |
| --- | --- | --- | --- | --- | --- | --- |
| **Intimate partner violence screening** | Screening for risk of intimate partner violence in all outpatients | Intimate partner violence (IPV) encompasses real or threatened psychologic, physical, or sexual harm by a current or former partner or spouse. IPV occurs among all gender identities and sexual orientations, through is more common in female and sexual/gender minorities. Rates of intimate partner violence are higher in a family where a member has been deployed, and current prevalence among veterans (2012, via telephone survey) were around 19% in randomly sampled females. IPV has significant correlations with physical and mental health impacts, including mortality risk, with cumulative and compounding effects over time. | In women - USPSTF: "B" (moderate benefit).  In older or vulnerable adults - USPSTF: "I" (insufficient). | 135 | All US Veterans | ^83,84^ |
| **Liver cancer screening in high-risk patients** | Screening ultrasound every 6 months for patients at high-risk for liver cancer (e.g., known cirrhosis, alcohol use disorder) | Veteran populations have five times the incidence of hepatocellular carcinoma (HCC) compared with the general population, largely due to cirrhosis. Screening is associated with ~33% reduced mortality and nearly 2-fold greater odds of earlier tumor detection. | Am. Assoc. for Study of Liver Diseases: "Low" (conditional recommendation) | 0.27 | All US Veterans (select diagnoses) | ^85–89^ |
| **Lung cancer screening in at-risk patients** | Lung cancer screening discussion for patients ages 50-80y who used cigarettes in the past 15 years | Lung cancer is the leading cause of cancer-related death in the US. While prevention by reducing smoking exceeds the impact on reducing cancer mortality compared to screening, screening with low-dose CT scans has been shown to reduce the morbidity and mortality from lung cancer. In the National Lung Screening randomized trial, for those undergoing at least 1 screen, the number needed to screen to prevent one lung cancer death is 320, with an all-cause mortality reduction of 7% in high-risk patients. However, low-dose CT is sensitive and has a high rate of false-positive, noncancerous finding that can lead to further testing and procedures. Low-dose CT for screening is a recommendation by the USPSTF. | USPSTF: "B" (moderate benefit). | 0.63 | All US Veterans (age-specific) | ^89,90^ |

| **Healthcare Process** | **Description** | **Evidence discussion** | **Level of Evidence & Guideline Source** | **Prevalence (per 1,000)** | **Target Pop.** | **Source** |
| --- | --- | --- | --- | --- | --- | --- |
| **Meningococcal immunization in at-risk patients** | Meningococcal immunization in at-risk patients | The US incidence declined from 0.61 cases to 0.15 cases per 100,000 pre- and post-booster dose recommendations for meningococcal disease. While cases are rare as of 2015, 14.9% of cases were fatal, and infection is associated with serious long-term consequences. Adult dosing (after age 23y) of meningococcal vaccine focuses on immunodeficient groups, and those with increased exposure risk, including prior unimmunized or under immunized active-duty military recruits in congregate living situations. | Advisory Committee on Immunization Practices (ACIP): Recommended. | 0.003 | All US Veterans (select diagnoses) | ^91–93^ |
| **Military toxic exposures screening** | Screening and provider follow-up on potential exposure to hazardous chemicals and materials during military service. | Veterans may have been exposed to a variety of chemical, physical, and environmental hazards during military service. Screening for these exposures is part of a large systemic effort related to passage of the 2022 VA PACT Act (Sergeant First Class (SFC) Heath Robinson Honoring our Promise to Address Comprehensive Toxics (PACT) Act) to extend eligibility and benefits. The law codifies 23 medical conditions, primarily respiratory and malignancy, that are related to military service exposure, so there is a lower burden of proof for linking health conditions to military exposures. Within the VHA, providers and medical teams are encouraged to continue to care for conditions and symptoms within usual medical care. Toxic screening-positive patients are referred to the Benefits administration for ongoing evaluation of their service-connected disabilities and benefits. | No clinical practice guidelines. | 860 | All US Veterans | ^31,94,95^ |
| **Obese patients participating in weight management** | Obese/At-risk (BMI > 24.9) patients who participated in an evidence-based weight management program (MOVE!) without contraindications | Group or individual comprehensive lifestyle intervention including behavioral, dietary, and physical activity components for patients with overweight or obesity are a recommended offering (for in-person interventions) in VA/DOD Clinical Practice Guidelines. For patients with existing diabetes, 5% weight loss has benefits for control of diabetes and cardiovascular risk factors (American Diabetes Association, "B" (e.g., well-conducted cohort studies) grade evidence). | VA/DOD: "Strong for" (high confidence). | 127 | All US Veterans (select diagnoses) | ^96,97^ |
| **Healthcare Process** | **Description** | **Evidence discussion** | **Level of Evidence & Guideline Source** | **Prevalence (per 1,000)** | **Target Pop.** | **Source** |
| **Opioid use disorder: MAT** | Patients with opioid use disorder on medication-assisted treatment (MAT; opioid agonist or antagonist treatment or who had a visit to an opioid substitution clinic) | For patients with opioid use disorder (OUD, around 1% of Veterans in 2021), it is recommended to offer one of the following considering patient preferences: Buprenorphine/naloxone or Methadone in an Opioid Treatment Program. Medication for OUD reinforces abstinence and improves treatment retention. | VA/DOD: "Strong for" (high confidence). | 7.1 | All US Veterans (select diagnoses) | ^29,98^ |
| **Opioids: Narcotic safety** | Narcotic safety and side effect monitoring in patients on long-term (>/= 90d) narcotics with side effect evaluation, overdose education and prevention (naloxone prescription) | Veterans with opioid use disorder (around 1% of Veterans in 2021) are at high risk for overdose. Education can help prevent opioid overdose and administration of naloxone can reverse the effects of an overdose if given in a timely manner, helping to reduce morbidity and mortality. VHA has ongoing national efforts to increase opioid overdose education and naloxone distribution to patients with opioid use disorder. | No clinical practice guidelines. | 7.1 | All US Veterans (select diagnoses) | ^98^ |
| **Osteoporosis screening in women >/= 65y** | Osteoporosis screening test (e.g., DEXA) in eligible women 65-75y | The USPSTF recommends screening for osteoporosis with bone measurement testing to prevent fractures in women >/= 65 years, with a "B" level of evidence for the recommendation (moderate certainty of benefit). | USPSTF: "B" (moderate benefit). | 188 | Women US Veterans (age-specific) | ^99,100^ |
| **Pneumococcal immunization** | Pneumococcal immunization (PPSV23 or PCV15/20) in all eligible patients after age 60y or in younger patients at high-risk | Pneumococcal infections (e.g., pneumonia, meningitis) are an important cause of morbidity and mortality, especially among older adults. In meta-analyses including 64,500 individuals, PPSV23 vaccination reduced the risk of invasive pneumococcal disease ([OR] 0.18, 95% CI 0.10-0.31)), as well as invasive and noninvasive pneumococcal pneumonia. In cohort studies of older adults, community-acquired pneumonia was associated with a 6-fold risk of increased mortality and 16% lower quality-of-life in the post-discharge year among patients surviving hospitalization for community-acquired pneumonia, compared to non-diseased persons. | Advisory Committee on Immunization Practices (ACIP): Recommended. | 5.8 | All US Veterans (age-specific) | ^101–103^ |

| **Healthcare Process** | **Description** | **Evidence discussion** | **Level of Evidence & Guideline Source** | **Prevalence (per 1,000)** | **Target Pop.** | **Source** |
| --- | --- | --- | --- | --- | --- | --- |
| **PTSD screening** | PTSD screening in all outpatients | PTSD is the mental health disorder most commonly associated with combat, and rates in US Veterans exceed the US population. Screening for PTSD is an essential step in identifying and engaging veterans with PTSD. Early intervention is intended to prevent chronic PTSD and its complicating disorders and functional problems, including major depression, suicide, substance use disorder, family violence, and homelessness. Periodic screening using validated measures such as the Primary Care PTSD Screen (PC-PTSD) is a "weak" evidence-level recommendation by the VA/DOD Clinical Practice Guidelines. | VA/DOD: "Weak for" (suggested conditionally). | 59 | All US Veterans | ^104,105^ |
| **Sexual trauma screening** | Military sexual trauma screening in all outpatients | Overall, as many as 16% of military personnel and veterans report military sexual trauma (4% of men, 38% of women), including both sexual harassment and assault. Of these, around 14% specifically report assault (2% of men, 24% of women) and 31% report harassment (9% of men, 53% of women). | No clinical practice guidelines. | 157 | All US Veterans | ^106^ |
| **Statin therapy for high-risk patients** | High or moderate-dose statin therapy (or exemption if appropriate) for high-risk patients with cardiovascular disease or diabetes | Cardiovascular disease (CVD) is the leading cause of death in the US. Statins reduce cardiovascular risk; one study found a 12-25% increase in mortality risk with nonadherence to statins after an acute myocardial infarction. The risk of an adult with diabetes developing cardiovascular disease (CVD) is 2-4x higher, and subsequent risk of CVD-related death is 1.7x greater than adults without diabetes. | For secondary prevention - VA/DOD: "Strong for" (high confidence). For primary prevention - Am. Diabetes Assoc. / Am. College Cardiology: "A" (high quality). | 205 | All US Veterans (select diagnoses) | ^35,46^ |

| **Healthcare Process** | **Description** | **Evidence discussion** | **Level of Evidence & Guideline Source** | **Prevalence (per 1,000)** | **Target Pop.** | **Source** |
| --- | --- | --- | --- | --- | --- | --- |
| **Tdap immunization** | Tdap/Td immunization every 10 years, in all eligible patients (or exclusion if contraindication) | Tdap is a routine adult vaccine recommended by Advisory Committee on Immunization Practices. While clinical disease is rare in the US, immunity wanes after childhood vaccination, and adults are susceptible for disease re-emergence (e.g., only 30% of US adults >/= 60y are serologically immune to diphtheria). Tdap vaccination has reduced the number of cases dramatically since introduction in 1980. | Advisory Committee on Immunization Practices (ACIP): Recommended. | 0.053 | All US Veterans | ^107^ |
| **Tobacco cessation intervention** | Tobacco cessation intervention (advice to quit, strategies, discussion of FDA-approved medications) in regular smokers | For adults using tobacco, a combination of behavioral interventions plus pharmacotherapy is recommended to maximize quit rates. This is an "A" (high quality evidence, strong benefit) recommendation by the Department of Health and Human Services, American College of Cardiology/American Heart Association, and the USPSTF. | USPSTF: "A" (Strong benefit) | 330 | All US Veterans (select diagnoses) | ^108–110^ |
| **Tobacco use screening and evaluation of usage** | Tobacco use screening in all outpatients | Three out of 10 U.S. Veterans use tobacco. All patients should be asked if they use tobacco and should have their tobacco use status documented on a regular basis. Clinic screening systems, such as expanding the vital signs to include tobacco use status or the use of other reminder systems as chart stickers or computer prompts, significantly increase rates of clinician intervention. | USPSTF: "A" (Strong benefit) | 330 | All US Veterans | ^109,110^ |
| **Traumatic brain injury screening in at-risk patients** | Post 9/11-theater patients screened for traumatic brain injury | Traumatic Brain Injury (TBI) is a common injury found in service members serving in Post-9/11 theaters. Screening could contribute to ensuring that individuals with TBI are identified and treated. Sequalae of traumatic brain injury (TBI) can include sleep-wake disturbances, headache, and posttraumatic vertigo. While most symptoms and disability resolve shortly after injury, a minority of patients can have deficits persisting at a year or longer, and repeat injury can cause more severe and protracted prolonged cognitive deficits. Evaluating patients with symptoms potentially related to brain injury is a "strong" recommendation by VA/DOD Clinical Practice Guidelines. | VA/DOD: "Strong for" (high confidence). | 70 | Veterans with service post 9/11 | ^111,112^ |
| **Healthcare Process** | **Description** | **Evidence discussion** | **Level of Evidence & Guideline Source** | **Prevalence (per 1,000)** | **Target Pop.** | **Source** |
| **Urinary incontinence screening in older adults** | Annual screening for urinary incontinence in older adults >/= 75y | Urinary incontinence (UI) is a frequent dysfunction of older adults. Without treatment the likelihood of progression is great. | No clinical practice guidelines. | 167 | All US Veterans (age-specific) | ^113^ |

Reference sources appear at end of supplement.

**eTable 2. Potential criteria and uncertainty simulations around rankings.**

| **Criteria**  *(“To contribute towards outcome [at right], it is most important for care processes to…”)* | **Mortality** | | | **Function** | | | **Symptoms** | | |
| --- | --- | --- | --- | --- | --- | --- | --- | --- | --- |
|  | **Rank** | **% at Rank** | **% in Top 7** | **Rank** | **% at Rank** | **% in Top 7** | **Rank** | **% at Rank** | **% in Top 7** |
| Be directly actionable by clinicians/team | 1 | 54.9 | 71.2 | 1 | 41.0 | 97.6 | 1 | 30.4 | 96.7 |
| Reflect high-prevalence health issues (affecting the most patients possible) | 2 | 19.4 | 80.8 | 4 | 12.4 | 75.4 | 5 | 7.5 | 61.5 |
| Be highly valid (measuring what they are supposed to measure) | 3 | 14.3 | 75.1 | 6 | 9.9 | 59.2 | 3 | 22.6 | 91.9 |
| Be grounded in published, high-quality evidence | 4 | 10.8 | 67.8 | 7 (tie) | 11.4 | 65.9 | 4 | 14.4 | 82.1 |
| Be highly reliable (provides same results if measured repeatedly) | 5 | 9.6 | 64.2 | 10 | 18.8 | 31.9 | 9 | 17.0 | 50.7 |
| Advance safety and reduce harm to patients | 6 | 9.0 | 67.9 | 11 | 19.9 | 22.9 | 2 | 4.2 | 28.2 |
| Reflect longer-term health goals (e.g., outcome measures) | 7 | 6.9 | 49.7 | 2 | 18.6 | 91.3 | 12 | 7.9 | 9.2 |
| Contribute to timely, efficient, and accessible care | 8 | 11.4 | 57.9 | 5 | 9.6 | 64.3 | 8 | 12.0 | 52.1 |
| Be easy to complete as measurement tasks and/or measured via EHR data | 9 | 13.5 | 51.9 | 3 | 14.1 | 79.5 | 6 | 17.7 | 81.7 |
| Improve service effectiveness (optimize appropriate care, minimize waste/medical burden) | 10 | 9.7 | 58.1 | 12 | 4.9 | 16.6 | 7 | 12.5 | 60.7 |
| Reflect shorter-term goals (e.g., process measures) | 11 | 15.9 | 36.8 | 7 (tie) | 9.1 | 53.4 | 11 | 13.4 | 30.7 |
| Advance care that is equitable (accurate and fair across patient subgroups) | 12 | 3.3 | 28.8 | 7 (tie) | 11.3 | 64.4 | 9 | 15.0 | 39.9 |

**eTable 3. Per-criteria scores and rankings for healthcare processes for Mortality goal domain.**

| **Healthcare Process** | **Mortality** | | | | | | | | |
| --- | --- | --- | --- | --- | --- | --- | --- | --- | --- |
|  | **Advance safety** | **Ease of task** | **High-prev. issue** | **Action-ability** | **Evidence based** | **Long-term goal** | **Validity** | **Total** | **Rank** |
| Tobacco use screen/eval. of usage | 0.68 | 0.50 | 1.17 | 2.53 | 0.88 | 0.67 | 0.98 | 7.41 | 1 |
| Influenza immunization in pts. >/= 65y | 0.67 | 0.44 | 1.02 | 2.41 | 0.82 | 0.58 | 0.99 | 6.92 | 2 |
| Pneumococcal immunization | 0.61 | 0.45 | 1.05 | 2.47 | 0.79 | 0.58 | 0.94 | 6.90 | 3 |
| Diabetes: kidney health screening | 0.60 | 0.44 | 1.11 | 2.41 | 0.79 | 0.60 | 0.94 | 6.88 | 4 |
| HIV testing in average-risk patients | 0.61 | 0.48 | 0.93 | 2.45 | 0.79 | 0.60 | 0.95 | 6.82 | 5 |
| Diabetes: glucose control | 0.63 | 0.46 | 1.14 | 2.15 | 0.84 | 0.60 | 0.97 | 6.79 | 6 |
| Hepatitis B immuniz. in high-risk pts. | 0.65 | 0.46 | 0.88 | 2.47 | 0.84 | 0.55 | 0.94 | 6.78 | 7 |
| Depression screening | 0.65 | 0.45 | 1.11 | 2.28 | 0.77 | 0.58 | 0.89 | 6.73 | 8 |
| Diabetes: retinal screening | 0.51 | 0.42 | 0.97 | 2.53 | 0.79 | 0.56 | 0.92 | 6.70 | 9 |
| Influenza immunization in pts. < 65y | 0.61 | 0.46 | 0.99 | 2.47 | 0.73 | 0.53 | 0.89 | 6.69 | 10 |
| Tobacco cessation intervention | 0.63 | 0.40 | 1.08 | 2.03 | 0.88 | 0.63 | 0.94 | 6.59 | 11 |
| Housing / food insecurity screening | 0.63 | 0.41 | 1.08 | 2.34 | 0.64 | 0.62 | 0.86 | 6.59 | 12 |
| Fall screen/function assess, older pts. | 0.70 | 0.35 | 1.11 | 2.22 | 0.75 | 0.63 | 0.81 | 6.57 | 13 |
| Blood pressure control, hyperten. pts. | 0.51 | 0.48 | 1.11 | 2.15 | 0.82 | 0.60 | 0.86 | 6.53 | 14 |
| Diabetes: foot examination | 0.63 | 0.35 | 0.85 | 2.28 | 0.79 | 0.63 | 0.92 | 6.45 | 15 |
| Cervical cancer screen, eligible pts. | 0.58 | 0.40 | 0.85 | 2.28 | 0.84 | 0.52 | 0.97 | 6.42 | 16 |
| HPV immunization | 0.54 | 0.45 | 0.86 | 2.53 | 0.74 | 0.42 | 0.88 | 6.41 | 17 |
| Meningococcal immuniz., at-risk pts. | 0.54 | 0.45 | 0.82 | 2.34 | 0.75 | 0.53 | 0.89 | 6.33 | 18 |
| Blood pressure control in diabetes | 0.51 | 0.42 | 0.89 | 2.11 | 0.85 | 0.56 | 0.95 | 6.29 | 19 |
| Cholesterol monitoring, at-risk pts. | 0.47 | 0.40 | 0.97 | 2.45 | 0.65 | 0.53 | 0.81 | 6.28 | 20 |
| Breast cancer screen, eligible pts. | 0.54 | 0.44 | 0.96 | 2.28 | 0.64 | 0.50 | 0.92 | 6.28 | 21 |
| Beta blockers, prior myocardial infarct | 0.54 | 0.46 | 0.82 | 2.34 | 0.66 | 0.53 | 0.89 | 6.25 | 22 |
| Tdap immunization | 0.54 | 0.46 | 0.73 | 2.34 | 0.77 | 0.50 | 0.89 | 6.24 | 23 |
| Colorectal ca. screen, ave.-risk pts. | 0.56 | 0.42 | 0.96 | 2.13 | 0.81 | 0.49 | 0.85 | 6.22 | 24 |
| Osteoporosis screen, women >/= 65y | 0.54 | 0.47 | 0.78 | 2.36 | 0.65 | 0.51 | 0.92 | 6.22 | 25 |
| Opioids: narcotic safety | 0.65 | 0.42 | 0.89 | 2.15 | 0.77 | 0.56 | 0.71 | 6.15 | 26 |
| Opioid use disorder: MAT | 0.63 | 0.33 | 0.90 | 2.03 | 0.77 | 0.60 | 0.89 | 6.15 | 27 |
| Hepatitis C screen, ave.-risk pts. | 0.47 | 0.43 | 0.79 | 2.28 | 0.68 | 0.55 | 0.92 | 6.11 | 28 |
| Alcohol misuse/use d/o screen | 0.60 | 0.35 | 0.99 | 1.96 | 0.77 | 0.58 | 0.84 | 6.09 | 29 |
| Follow-up, positive suicide risk screen | 0.65 | 0.40 | 0.93 | 2.03 | 0.66 | 0.57 | 0.81 | 6.05 | 30 |
| Abd. aorta aneurysm scrn., at-risk pt. | 0.49 | 0.40 | 0.76 | 2.34 | 0.60 | 0.53 | 0.89 | 6.01 | 31 |
| Chlamydia screen, young women | 0.60 | 0.36 | 0.82 | 2.15 | 0.68 | 0.50 | 0.89 | 6.00 | 32 |
| Lung cancer screen, at-risk pts. | 0.51 | 0.43 | 0.78 | 2.36 | 0.65 | 0.47 | 0.78 | 5.98 | 33 |
| Statin therapy, high-risk pts. | 0.47 | 0.40 | 0.97 | 2.11 | 0.77 | 0.51 | 0.75 | 5.97 | 34 |
| Medication reconciliation | 0.63 | 0.32 | 0.93 | 2.11 | 0.59 | 0.53 | 0.81 | 5.93 | 35 |
| Intimate partner violence screening | 0.68 | 0.34 | 0.96 | 1.84 | 0.71 | 0.57 | 0.79 | 5.88 | 36 |
| Liver cancer screen, at-risk pts. | 0.58 | 0.40 | 0.76 | 2.15 | 0.64 | 0.52 | 0.81 | 5.86 | 37 |
| **Healthcare Process** | **Mortality** | | | | | | | | |
|  | **Advance safety** | **Ease of task** | **High-prev. issue** | **Action-ability** | **Evidence based** | **Long-term goal** | **Validity** | **Total** | **Rank** |
| High-risk pts. referred, palliative care | 0.54 | 0.42 | 0.82 | 2.28 | 0.50 | 0.60 | 0.68 | 5.83 | 38 |
| Heart failure: wt. monitoring edu. | 0.56 | 0.40 | 0.90 | 1.90 | 0.64 | 0.50 | 0.81 | 5.72 | 39 |
| Heart failure: ACE or ARB use | 0.47 | 0.40 | 0.78 | 2.03 | 0.74 | 0.51 | 0.75 | 5.66 | 40 |
| Urinary incontinence in older adults | 0.57 | 0.37 | 0.84 | 1.93 | 0.60 | 0.57 | 0.75 | 5.64 | 41 |
| Annual suicide risk screening | 0.58 | 0.40 | 0.90 | 1.96 | 0.51 | 0.58 | 0.69 | 5.62 | 42 |
| Alcohol misuse/use d/o intervention | 0.54 | 0.32 | 0.89 | 1.86 | 0.71 | 0.53 | 0.75 | 5.59 | 43 |
| Follow-up, pos. PTSD/depress. scrn | 0.51 | 0.28 | 0.97 | 1.94 | 0.59 | 0.51 | 0.71 | 5.52 | 44 |
| PTSD screening | 0.58 | 0.36 | 0.90 | 1.71 | 0.68 | 0.52 | 0.71 | 5.46 | 45 |
| Depression monitoring | 0.54 | 0.35 | 0.86 | 1.77 | 0.62 | 0.47 | 0.71 | 5.31 | 46 |
| Obese pts. in weight management | 0.42 | 0.32 | 0.86 | 1.77 | 0.65 | 0.47 | 0.64 | 5.12 | 47 |
| Diabetes: glucose monitoring | 0.47 | 0.45 | 0.78 | 1.77 | 0.56 | 0.42 | 0.64 | 5.09 | 48 |
| Hepatitis B immuniz., ave.-risk pts. | 0.40 | 0.35 | 0.47 | 1.94 | 0.56 | 0.38 | 0.75 | 4.84 | 49 |
| Traumatic brain injury scrn., at-risk pt. | 0.40 | 0.28 | 0.70 | 1.69 | 0.50 | 0.42 | 0.71 | 4.70 | 50 |
| Sexual trauma screening | 0.42 | 0.27 | 0.74 | 1.60 | 0.44 | 0.47 | 0.61 | 4.55 | 51 |
| Embedded fragment screening | 0.37 | 0.30 | 0.61 | 1.71 | 0.40 | 0.32 | 0.56 | 4.26 | 52 |
| Military toxic exposures screening | 0.46 | 0.29 | 0.67 | 1.20 | 0.31 | 0.37 | 0.56 | 3.85 | 53 |

**eTable 4. Per-criteria scores and rankings for healthcare processes for Function goal domain.**

| **Care process** | **Function** | | | | | | | | |
| --- | --- | --- | --- | --- | --- | --- | --- | --- | --- |
|  | **Advance safety** | **Ease of task** | **High-prev. issue** | **Action-ability** | **Evidence based** | **Long-term goal** | **Validity** | **Total** | **Rank** |
| Tobacco use screen/eval. of usage | 0.33 | 1.10 | 1.00 | 1.64 | 0.74 | 1.26 | 0.79 | 6.86 | 1 |
| Diabetes: kidney health screening | 0.29 | 0.96 | 0.95 | 1.56 | 0.67 | 1.13 | 0.76 | 6.32 | 2 |
| Pneumococcal immunization | 0.3 | 0.99 | 0.90 | 1.60 | 0.67 | 1.10 | 0.76 | 6.31 | 3 |
| HIV testing in average-risk patients | 0.29 | 1.06 | 0.80 | 1.59 | 0.67 | 1.13 | 0.77 | 6.31 | 4 |
| Diabetes: glucose control | 0.31 | 1.02 | 0.98 | 1.39 | 0.70 | 1.13 | 0.78 | 6.31 | 5 |
| Influenza immunization in pts. >/= 65y | 0.32 | 0.96 | 0.88 | 1.56 | 0.68 | 1.10 | 0.80 | 6.31 | 6 |
| Depression screening | 0.31 | 0.99 | 0.95 | 1.48 | 0.65 | 1.10 | 0.72 | 6.20 | 7 |
| Hepatitis B vaccine in high-risk pts. | 0.31 | 1.02 | 0.75 | 1.60 | 0.70 | 1.04 | 0.76 | 6.18 | 8 |
| Blood pressure control, hyperten. pts. | 0.25 | 1.05 | 0.95 | 1.39 | 0.68 | 1.13 | 0.70 | 6.15 | 9 |
| Tobacco cessation intervention | 0.31 | 0.88 | 0.93 | 1.31 | 0.74 | 1.20 | 0.76 | 6.12 | 10 |
| Influenza immunization in pts. < 65y | 0.30 | 1.02 | 0.85 | 1.60 | 0.61 | 1.01 | 0.72 | 6.10 | 11 |
| Diabetes: retinal screening | 0.25 | 0.92 | 0.83 | 1.64 | 0.67 | 1.05 | 0.74 | 6.09 | 12 |
| Housing / food insecurity screening | 0.31 | 0.91 | 0.93 | 1.52 | 0.54 | 1.17 | 0.70 | 6.05 | 13 |
| Fall screen/function assess, older pts. | 0.34 | 0.77 | 0.95 | 1.44 | 0.63 | 1.20 | 0.66 | 5.98 | 14 |
| Diabetes: foot examination | 0.31 | 0.77 | 0.73 | 1.48 | 0.67 | 1.20 | 0.74 | 5.88 | 15 |
| Blood pressure control in diabetes | 0.25 | 0.92 | 0.77 | 1.37 | 0.72 | 1.05 | 0.77 | 5.83 | 16 |
| Meningococcal immuniz., at-risk pts. | 0.26 | 0.99 | 0.70 | 1.52 | 0.63 | 1.01 | 0.72 | 5.83 | 17 |
| Cervical cancer screen, eligible pts. | 0.28 | 0.88 | 0.73 | 1.48 | 0.70 | 0.98 | 0.78 | 5.82 | 18 |
| Beta blockers, prior myocardial infarct | 0.26 | 1.02 | 0.70 | 1.52 | 0.56 | 1.01 | 0.72 | 5.78 | 19 |
| HPV immunization | 0.26 | 0.99 | 0.73 | 1.64 | 0.62 | 0.80 | 0.71 | 5.75 | 20 |
| Breast cancer screen, eligible pts. | 0.26 | 0.96 | 0.83 | 1.48 | 0.54 | 0.95 | 0.74 | 5.75 | 21 |
| Tdap immunization | 0.26 | 1.02 | 0.63 | 1.52 | 0.65 | 0.95 | 0.72 | 5.73 | 22 |
| Cholesterol monitoring, at-risk pts. | 0.23 | 0.88 | 0.83 | 1.59 | 0.54 | 1.01 | 0.66 | 5.73 | 23 |
| Osteoporosis screen, women >/= 65y | 0.26 | 1.03 | 0.67 | 1.53 | 0.54 | 0.97 | 0.74 | 5.73 | 24 |
| Colorectal ca. screen, ave.-risk pts. | 0.27 | 0.92 | 0.82 | 1.38 | 0.68 | 0.93 | 0.69 | 5.70 | 25 |
| Hepatitis C screen, ave.-risk pts. | 0.23 | 0.94 | 0.68 | 1.48 | 0.57 | 1.04 | 0.74 | 5.67 | 26 |
| Opioids: narcotic safety | 0.32 | 0.92 | 0.77 | 1.39 | 0.64 | 1.05 | 0.57 | 5.66 | 27 |
| Opioid use disorder: MAT | 0.31 | 0.72 | 0.78 | 1.31 | 0.65 | 1.13 | 0.72 | 5.61 | 28 |
| Alcohol misuse/use d/o screen | 0.29 | 0.77 | 0.85 | 1.27 | 0.65 | 1.10 | 0.68 | 5.61 | 29 |
| Follow-up, positive suicide risk screen | 0.31 | 0.88 | 0.8 | 1.31 | 0.56 | 1.07 | 0.66 | 5.59 | 30 |
| Statin therapy for high-risk patients | 0.23 | 0.88 | 0.83 | 1.37 | 0.64 | 0.97 | 0.60 | 5.52 | 31 |
| Abd. aorta aneurysm scrn., at-risk pt. | 0.24 | 0.88 | 0.65 | 1.52 | 0.50 | 1.01 | 0.72 | 5.51 | 32 |
| High-risk pts. referred, palliative care | 0.26 | 0.92 | 0.70 | 1.48 | 0.42 | 1.13 | 0.55 | 5.45 | 33 |
| Lung cancer screen , at-risk pts. | 0.25 | 0.95 | 0.67 | 1.53 | 0.54 | 0.88 | 0.63 | 5.45 | 34 |
| Chlamydia screen, young women | 0.29 | 0.80 | 0.70 | 1.39 | 0.57 | 0.95 | 0.72 | 5.42 | 35 |
| Intimate partner violence screening | 0.33 | 0.74 | 0.83 | 1.19 | 0.59 | 1.07 | 0.64 | 5.39 | 36 |
| Liver cancer screen, at-risk pts. | 0.28 | 0.88 | 0.65 | 1.39 | 0.54 | 0.98 | 0.66 | 5.37 | 37 |
| **Healthcare Process** | **Function** | | | | | | | | |
|  | **Advance safety** | **Ease of task** | **High-prev. issue** | **Action-ability** | **Evidence based** | **Long-term goal** | **Validity** | **Total** | **Rank** |
| Medication reconciliation | 0.31 | 0.70 | 0.80 | 1.37 | 0.49 | 1.01 | 0.66 | 5.33 | 38 |
| Heart failure: wt. monitoring edu. | 0.27 | 0.88 | 0.78 | 1.23 | 0.54 | 0.95 | 0.66 | 5.29 | 39 |
| Annual suicide risk screening | 0.28 | 0.88 | 0.78 | 1.27 | 0.43 | 1.10 | 0.55 | 5.29 | 40 |
| Heart failure: ACE or ARB use | 0.23 | 0.88 | 0.67 | 1.31 | 0.62 | 0.97 | 0.60 | 5.27 | 41 |
| Urinary incontinence in older adults | 0.28 | 0.81 | 0.72 | 1.25 | 0.50 | 1.08 | 0.61 | 5.25 | 42 |
| Alcohol misuse/use d/o intervention | 0.26 | 0.70 | 0.77 | 1.20 | 0.59 | 1.01 | 0.60 | 5.13 | 43 |
| PTSD screening | 0.28 | 0.80 | 0.78 | 1.11 | 0.57 | 0.98 | 0.57 | 5.08 | 44 |
| Follow-up, pos. PTSD/depress. scrn | 0.25 | 0.62 | 0.83 | 1.26 | 0.49 | 0.97 | 0.57 | 5.00 | 45 |
| Depression monitoring | 0.26 | 0.77 | 0.73 | 1.15 | 0.52 | 0.88 | 0.57 | 4.89 | 46 |
| Diabetes: glucose monitoring | 0.23 | 0.99 | 0.67 | 1.15 | 0.47 | 0.80 | 0.52 | 4.82 | 47 |
| Obese pts. in weight management | 0.20 | 0.70 | 0.73 | 1.15 | 0.54 | 0.88 | 0.52 | 4.73 | 48 |
| Hepatitis B immuniz., ave.-risk pts. | 0.19 | 0.77 | 0.40 | 1.26 | 0.47 | 0.71 | 0.60 | 4.40 | 49 |
| Traumatic brain injury scrn., at-risk pt. | 0.19 | 0.62 | 0.60 | 1.09 | 0.42 | 0.80 | 0.57 | 4.30 | 50 |
| Sexual trauma screening | 0.20 | 0.59 | 0.63 | 1.04 | 0.37 | 0.88 | 0.49 | 4.21 | 51 |
| Embedded fragment screening | 0.18 | 0.66 | 0.53 | 1.11 | 0.33 | 0.60 | 0.45 | 3.85 | 52 |
| Military toxic exposures screening | 0.22 | 0.63 | 0.58 | 0.78 | 0.26 | 0.69 | 0.45 | 3.61 | 53 |

**eTable 5. Per-criteria scores and rankings for healthcare processes for Symptom Relief goal domain.**

| **Healthcare Process** | **Symptom Relief** | | | | | | | | |
| --- | --- | --- | --- | --- | --- | --- | --- | --- | --- |
|  | **Advance safety** | **Ease of task** | **High-prev. issue** | **Action-ability** | **Evidence based** | **Long-term goal** | **Validity** | **Total** | **Rank** |
| Tobacco use screen/eval. of usage | 1.29 | 0.88 | 0.95 | 1.52 | 1.03 | 0.20 | 1.26 | 7.13 | 1 |
| Influenza immunization in pts. >/= 65y | 1.26 | 0.77 | 0.83 | 1.44 | 0.96 | 0.18 | 1.27 | 6.71 | 2 |
| Diabetes: glucose control | 1.20 | 0.82 | 0.93 | 1.29 | 0.98 | 0.18 | 1.24 | 6.63 | 3 |
| Pneumococcal immunization | 1.16 | 0.79 | 0.86 | 1.48 | 0.93 | 0.18 | 1.20 | 6.60 | 4 |
| Hepatitis B immuniz. in high-risk pts. | 1.23 | 0.82 | 0.71 | 1.48 | 0.98 | 0.17 | 1.20 | 6.59 | 5 |
| Diabetes: kidney health screening | 1.13 | 0.77 | 0.90 | 1.44 | 0.93 | 0.18 | 1.20 | 6.56 | 6 |
| HIV testing in average-risk patients | 1.15 | 0.85 | 0.76 | 1.47 | 0.93 | 0.18 | 1.21 | 6.56 | 7 |
| Depression screening | 1.23 | 0.79 | 0.90 | 1.37 | 0.90 | 0.18 | 1.14 | 6.51 | 8 |
| Tobacco cessation intervention | 1.20 | 0.71 | 0.88 | 1.21 | 1.03 | 0.19 | 1.20 | 6.42 | 9 |
| Influenza immunization in pts. < 65y | 1.16 | 0.82 | 0.81 | 1.48 | 0.85 | 0.16 | 1.14 | 6.42 | 10 |
| Diabetes: retinal screening | 0.98 | 0.74 | 0.79 | 1.52 | 0.93 | 0.17 | 1.17 | 6.29 | 11 |
| Fall screen/function assess, older pts. | 1.33 | 0.62 | 0.90 | 1.33 | 0.88 | 0.19 | 1.04 | 6.29 | 12 |
| Housing and food insecurity screening | 1.20 | 0.73 | 0.88 | 1.40 | 0.75 | 0.19 | 1.11 | 6.25 | 13 |
| Blood pressure control, hyperten. pts. | 0.96 | 0.84 | 0.90 | 1.29 | 0.96 | 0.18 | 1.11 | 6.24 | 14 |
| Cervical cancer screen, eligible pts. | 1.10 | 0.71 | 0.69 | 1.37 | 0.98 | 0.16 | 1.24 | 6.23 | 15 |
| Diabetes: foot examination | 1.20 | 0.62 | 0.69 | 1.37 | 0.93 | 0.19 | 1.17 | 6.16 | 16 |
| HPV immunization | 1.02 | 0.79 | 0.70 | 1.52 | 0.86 | 0.13 | 1.13 | 6.14 | 17 |
| Blood pressure control in diabetes | 0.98 | 0.74 | 0.73 | 1.26 | 1.00 | 0.17 | 1.21 | 6.08 | 18 |
| Meningococcal immuniz. , at-risk pts. | 1.03 | 0.79 | 0.67 | 1.40 | 0.88 | 0.16 | 1.14 | 6.07 | 19 |
| Colorectal ca. screen, ave.-risk pts. | 1.06 | 0.74 | 0.78 | 1.27 | 0.95 | 0.15 | 1.09 | 6.05 | 20 |
| Tdap immunization | 1.03 | 0.82 | 0.59 | 1.40 | 0.90 | 0.15 | 1.14 | 6.04 | 21 |
| Breast cancer screen, eligible pts. | 1.03 | 0.77 | 0.78 | 1.37 | 0.75 | 0.15 | 1.17 | 6.02 | 22 |
| Beta blockers, prior myocardial infarct | 1.03 | 0.82 | 0.67 | 1.40 | 0.77 | 0.16 | 1.14 | 5.99 | 23 |
| Osteoporosis screen, women >/= 65y | 1.02 | 0.82 | 0.63 | 1.42 | 0.76 | 0.15 | 1.17 | 5.97 | 24 |
| Opioids: narcotic safety | 1.24 | 0.74 | 0.73 | 1.29 | 0.90 | 0.17 | 0.91 | 5.97 | 25 |
| Opioid use disorder: MAT | 1.20 | 0.57 | 0.74 | 1.21 | 0.90 | 0.18 | 1.14 | 5.94 | 26 |
| Follow-up, positive suicide risk screen | 1.23 | 0.71 | 0.76 | 1.21 | 0.77 | 0.17 | 1.04 | 5.90 | 27 |
| Alcohol misuse/use d/o screen | 1.13 | 0.62 | 0.81 | 1.18 | 0.90 | 0.18 | 1.07 | 5.88 | 28 |
| Chlamydia screen, young women | 1.13 | 0.64 | 0.67 | 1.29 | 0.80 | 0.15 | 1.14 | 5.81 | 29 |
| Cholesterol monitoring, at-risk pts. | 0.89 | 0.71 | 0.79 | 1.47 | 0.76 | 0.16 | 1.04 | 5.81 | 30 |
| Hepatitis C screen, ave.-risk pts. | 0.90 | 0.75 | 0.64 | 1.37 | 0.80 | 0.17 | 1.17 | 5.79 | 31 |
| Intimate partner violence screening | 1.30 | 0.60 | 0.78 | 1.10 | 0.83 | 0.17 | 1.01 | 5.78 | 32 |
| Lung cancer scrning in at-risk patients | 0.98 | 0.77 | 0.63 | 1.42 | 0.76 | 0.14 | 1.00 | 5.68 | 33 |
| Medication reconciliation | 1.2 | 0.56 | 0.76 | 1.26 | 0.69 | 0.16 | 1.04 | 5.67 | 34 |
| Lung cancer screen, at-risk pts. | 1.1 | 0.71 | 0.62 | 1.29 | 0.75 | 0.16 | 1.04 | 5.65 | 35 |
| Abd. aorta aneurysm scrn., at-risk pt. | 0.93 | 0.71 | 0.62 | 1.40 | 0.70 | 0.16 | 1.14 | 5.65 | 36 |
| Statin therapy, high-risk pts. | 0.89 | 0.71 | 0.79 | 1.26 | 0.90 | 0.15 | 0.95 | 5.65 | 37 |
| **Healthcare Process** | **Symptom Relief** | | | | | | | | |
|  | **Advance safety** | **Ease of task** | **High-prev. issue** | **Action-ability** | **Evidence based** | **Long-term goal** | **Validity** | **Total** | **Rank** |
| Heart failure: wt. monitoring edu. | 1.06 | 0.71 | 0.74 | 1.14 | 0.75 | 0.15 | 1.04 | 5.58 | 38 |
| High-risk pts. referred, palliative care | 1.02 | 0.74 | 0.67 | 1.37 | 0.59 | 0.18 | 0.87 | 5.42 | 39 |
| Urinary incontinence in older adults | 1.09 | 0.65 | 0.68 | 1.15 | 0.70 | 0.17 | 0.96 | 5.42 | 40 |
| Heart failure: ACE or ARB use | 0.89 | 0.71 | 0.63 | 1.21 | 0.86 | 0.15 | 0.95 | 5.41 | 41 |
| Annual suicide risk screening | 1.10 | 0.71 | 0.74 | 1.18 | 0.59 | 0.18 | 0.88 | 5.36 | 42 |
| Alcohol misuse/use d/o intervention | 1.02 | 0.56 | 0.73 | 1.11 | 0.83 | 0.16 | 0.95 | 5.36 | 43 |
| PTSD screening | 1.10 | 0.64 | 0.74 | 1.02 | 0.79 | 0.16 | 0.91 | 5.35 | 44 |
| Follow-up, pos. PTSD/depress. scrn | 0.98 | 0.50 | 0.79 | 1.16 | 0.69 | 0.15 | 0.91 | 5.18 | 45 |
| Depression monitoring | 1.02 | 0.62 | 0.70 | 1.06 | 0.72 | 0.14 | 0.91 | 5.17 | 46 |
| Diabetes: glucose monitoring | 0.89 | 0.79 | 0.63 | 1.06 | 0.65 | 0.13 | 0.82 | 4.98 | 47 |
| Obese pts. in weight management | 0.80 | 0.56 | 0.70 | 1.06 | 0.76 | 0.14 | 0.82 | 4.84 | 48 |
| Hepatitis B immuniz., ave.-risk pts. | 0.75 | 0.62 | 0.38 | 1.16 | 0.65 | 0.11 | 0.95 | 4.64 | 49 |
| Traumatic brain injury scrn., at-risk pt. | 0.75 | 0.50 | 0.57 | 1.01 | 0.59 | 0.13 | 0.91 | 4.46 | 50 |
| Sexual trauma screening | 0.80 | 0.47 | 0.60 | 0.96 | 0.52 | 0.14 | 0.78 | 4.27 | 51 |
| Embedded fragment screening | 0.70 | 0.53 | 0.50 | 1.02 | 0.46 | 0.10 | 0.72 | 4.03 | 52 |
| Military toxic exposures screening | 0.86 | 0.51 | 0.55 | 0.72 | 0.36 | 0.11 | 0.72 | 3.83 | 53 |

**Supplementary Material References**

1.	Boulkedid R, Abdoul H, Loustau M, Sibony O, Alberti C. Using and reporting the Delphi method for selecting healthcare quality indicators: A systematic review. *PLoS ONE*. 2011;6(6):e20476. doi:10.1371/journal.pone.0020476

2.	Morse JM. Determining sample size. *Qual Health Res*. 2000;10(1):3-5. doi:10.1177/104973200129118183

3.	Chang ET, Newberry S, Rubenstein LV, Motala A, Booth MJ, Shekelle PG. Quality Measures for Patients at Risk of Adverse Outcomes in the Veterans Health Administration: Expert Panel Recommendations. *JAMA Netw Open*. 2022;5(8):e2224938. doi:10.1001/jamanetworkopen.2022.24938

4.	Fried TR. Health outcome prioritization as a tool for decision making among older persons with multiple chronic conditions. *Arch Intern Med*. 2011;171(20):1856. doi:10.1001/archinternmed.2011.424

5.	ACOVE Quality Indicators. *Ann Intern Med*. 2001;135(8_Part_2):653. doi:10.7326/0003-4819-135-8_Part_2-200110161-00004

6.	Shiyanbola OO, Mort JR. Patients’ perceived value of pharmacy quality measures: a mixed-methods study. *BMJ Open*. 2015;5(1):e006086-e006086. doi:10.1136/bmjopen-2014-006086

7.	Higashi T, Shekelle PG, Adams JL, et al. Quality of Care Is Associated with Survival in Vulnerable Older Patients. *Ann Intern Med*. 2005;143(4):274. doi:10.7326/0003-4819-143-4-200508160-00008

8.	Bayliss EA, McQuillan DB, Ellis JL, et al. Using Electronic Health Record Data to Measure Care Quality for Individuals with Multiple Chronic Medical Conditions. *J Am Geriatr Soc*. 2016;64(9):1839-1844. doi:10.1111/jgs.14248

9.	Prinsen CAC, Vohra S, Rose MR, et al. How to select outcome measurement instruments for outcomes included in a “Core Outcome Set” – a practical guideline. *Trials*. 2016;17(1):449. doi:10.1186/s13063-016-1555-2

10.	Shekelle PG. Assessing Care of Vulnerable Elders: Methods for Developing Quality Indicators. *Ann Intern Med*. 2001;135(8_Part_2):647. doi:10.7326/0003-4819-135-8_Part_2-200110161-00003

11.	Six Domains of Health Care Quality. Accessed April 23, 2020. http://www.ahrq.gov/talkingquality/measures/six-domains.html

12.	Groothuis-Oudshoorn C, Broekhuizen H, van Til JA. Dealing with uncertainty in the analysis and reporting of MCDA. In: Thokala P, Baltussen R, Marsh K, Goetghebeur M, eds. *Multi-Criteria Decision Analysis to Support Healthcare Decisions*. 1st ed. Springer International Publishing; 2017:67-85.

13.	*Honoring Our PACT Act of 2022*.; 2022:59. Accessed April 21, 2025. https://www.congress.gov/117/plaws/publ168/PLAW-117publ168.pdf

14.	Cohen J. A coefficient of agreement for nominal scales. *Educ Psychol Meas*. 1960;20(1):37-46.

15.	Aschmann HE, Puhan MA, Robbins CW, et al. Outcome preferences of older people with multiple chronic conditions and hypertension: a cross-sectional survey using best-worst scaling. *Health Qual Life Outcomes*. 2019;17(1):186. doi:10.1186/s12955-019-1250-6

16.	Tinetti ME, Costello DM, Naik AD, et al. Outcome Goals and Health Care Preferences of Older Adults With Multiple Chronic Conditions. *JAMA Netw Open*. 2021;4(3):e211271. doi:10.1001/jamanetworkopen.2021.1271

17.	Tinetti ME, McAvay GJ, Fried TR, et al. Development of a Tool for Eliciting Patient Priority from Among Competing Cardiovascular Disease, Medication-Symptoms, and Fall Injury Outcomes. *J Am Geriatr Soc*. 2008;56(4):730-736. doi:10.1111/j.1532-5415.2007.01627.x

18.	Sathanapally H, Sidhu M, Fahami R, et al. Priorities of patients with multimorbidity and of clinicians regarding treatment and health outcomes: a systematic mixed studies review. *BMJ Open*. 2020;10(2):e033445. doi:10.1136/bmjopen-2019-033445

19.	Smith SM, Wallace E, Salisbury C, Sasseville M, Bayliss E, Fortin M. A core outcome set for multimorbidity research (COSmm). *Ann Fam Med*. 2018;16(2):132-138. doi:10.1370/afm.2178

20.	Fihn SD, Francis J, Clancy C, et al. Insights from advanced analytics at the Veterans Health Administration. *Health Aff (Millwood)*. 2014;33(7):1203-1211. doi:10.1377/hlthaff.2014.0054

21.	Prenovost KM, Fihn SD, Maciejewski ML, Nelson K, Vijan S, Rosland AM. Using item response theory with health system data to identify latent groups of patients with multiple health conditions. *PLOS ONE*. 2018;13(11):e0206915. doi:10.1371/journal.pone.0206915

22.	Zulman DM, Asch SM, Martins SB, Kerr EA, Hoffman BB, Goldstein MK. Quality of care for patients with multiple chronic conditions: the role of comorbidity interrelatedness. *J Gen Intern Med*. 2014;29(3):529-537. doi:10.1007/s11606-013-2616-9

23.	Marsh K, IJzerman M, Thokala P, et al. Multiple criteria decision analysis for health care decision making—emerging good practices: Report 2 of the ISPOR MCDA Emerging Good Practices Task Force. *Value Health*. 2016;19(2):125-137. doi:10.1016/j.jval.2015.12.016

24.	Briggs AH, Weinstein MC, Fenwick EAL, et al. Model parameter estimation and uncertainty analysis: a report of the ISPOR-SMDM Modeling Good Research Practices Task Force Working Group-6. *Med Decis Mak Int J Soc Med Decis Mak*. 2012;32(5):722-732. doi:10.1177/0272989X12458348

25.	Briggs AH, Claxton K, Sculpher MJ. *Decision Modelling for Health Economic Evaluation*. Oxford University Press; 2006.

26.	Filipkowski AM, Kundu S, Eden SK, et al. Association of HIV infection and incident abdominal aortic aneurysm among 143,001 Veterans. *Circulation*. 2023;148(2):135-143. doi:10.1161/CIRCULATIONAHA.122.063040

27.	Lee ES, Chun KC, Gupta A, et al. Costs of abdominal aortic aneurysm care at a regional Veterans Affairs medical center with the implementation of an abdominal aortic aneurysm screening program. *J Vasc Surg*. 2022;75(4):1253-1259. doi:10.1016/j.jvs.2021.10.006

28.	US Preventive Services Task Force, Owens DK, Davidson KW, et al. Screening for abdominal aortic aneurysm: US Preventive Services Task Force recommendation statement. *JAMA*. 2019;322(22):2211. doi:10.1001/jama.2019.18928

29.	Department of Veterans Affairs, Department of Defense. *VA/DoD Clinical Practice Guideline for the Management of Substance Use Disorders*.; 2015. https://www.healthquality.va.gov/guidelines/mh/sud/vadodsudcpgrevised22216.pdf

30.	Jonas DE, Garbutt JC, Amick HR, et al. Behavioral counseling after screening for alcohol misuse in primary care: A systematic review and meta-analysis for the U.S. Preventive Services Task Force. *Ann Intern Med*. 2012;157(9):645-654. doi:10.7326/0003-4819-157-9-201211060-00544

31.	Hynes DM, Edwards S, Hickok A, et al. Veterans’ use of Veterans Health Administration primary care in an era of expanding choice. *Med Care*. 2021;59:S292. doi:10.1097/MLR.0000000000001554

32.	US Preventive Services Task Force, Curry SJ, Krist AH, et al. Screening and behavioral counseling interventions to reduce unhealthy alcohol use in adolescents and adults: US Preventive Services Task Force recommendation statement. *JAMA*. 2018;320(18):1899. doi:10.1001/jama.2018.16789

33.	Department of Veterans Affairs, Department of Defense. *VA/DoD Clinical Practice Guideline for Assessment and Management of Patients at Risk for Suicide*.; 2024. https://www.healthquality.va.gov/guidelines/MH/srb/VADOD-CPG-Suicide-Risk-Full-CPG-2024_Final_508.pdf

34.	Department of Veterans Affairs. *National Veteran Suicide Prevention Annual Report*.; 2021.

35.	Hinojosa R. Cardiovascular disease among United States military veterans: Evidence of a waning healthy soldier effect using the National Health Interview Survey - Ramon Hinojosa, 2020. *Sage*. 2018;16(1). doi:https://doi.org/10.1177/1742395318785237

36.	Stone NJ, Robinson JG, Lichtenstein AH, et al. 2013 ACC/AHA guideline on the treatment of blood cholesterol to reduce atherosclerotic cardiovascular risk in adults: A report of the American College of Cardiology/American Heart Association Task Force on practice guidelines. *Circulation*. 2014;129(25_suppl_2). doi:10.1161/01.cir.0000437738.63853.7a

37.	Virani SS, Newby LK, Arnold SV, et al. 2023 AHA/ACC/ACCP/ASPC/NLA/PCNA guideline for the management of patients with chronic coronary disease: A report of the American Heart Association/American College of Cardiology Joint Committee on Clinical Practice Guidelines. *Circulation*. 2023;148(9). doi:10.1161/CIR.0000000000001168

38.	Liu Y, Sayam S, Shao X, et al. Prevalence of and trends in diabetes among Veterans, United States, 2005-2014. *Prev Chronic Dis*. 2017;14:E135. doi:10.5888/pcd14.170230

39.	Passarella P, Kiseleva TA, Valeeva FV, Gosmanov AR. Hypertension management in diabetes: 2018 update. *Diabetes Spectr*. 2018;31(3):218-224. doi:10.2337/ds17-0085

40.	Oeffinger KC, Fontham ETH, Etzioni R, et al. Breast cancer screening for women at average risk: 2015 Guideline update from the American Cancer Society. *JAMA*. 2015;314(15):1599. doi:10.1001/jama.2015.12783

41.	US Preventive Services Task Force. Screening for breast cancer: US Preventive Services Task Force recommendation statement. *JAMA*. 2024;331(22):1918-1930. doi:10.1001/jama.2024.5534

42.	US Preventive Services Task Force. Screening for cervical cancer: US Preventive Services Task Force recommendation statement. *JAMA*. 2018;320(7):674-686. doi:10.1001/jama.2018.10897

43.	Keddem S, Maier M, Gardella C, et al. Gonorrhea and chlamydia testing and case rates among women Veterans in the Veterans Health Administration. *J Gen Intern Med*. 2022;37(Suppl 3):706-713. doi:10.1007/s11606-022-07578-2

44.	US Preventive Services Task Force. Screening for chlamydia and gonorrhea: US Preventive Services Task Force recommendation statement. *JAMA*. 2021;326(10):949-956. doi:10.1001/jama.2021.14081

45.	American Diabetes Association Professional Practice Committee, ElSayed NA, Aleppo G, et al. Cardiovascular disease and risk management: Standards of care in diabetes—2024. *Diabetes Care*. 2024;47(Supplement_1):S179-S218. doi:10.2337/dc24-S010

46.	Department of Veterans Affairs, Department of Defense. *VA/DoD Clinical Practice Guideline for the Management of Dyslipidemia for Cardiovascular Risk Reduction*.; 2020. https://www.healthquality.va.gov/guidelines/CD/lipids/VADODDyslipidemiaCPG5087212020.pdf

47.	Grundy SM, Stone NJ, Bailey AL, et al. 2018 AHA/ACC/AACVPR/AAPA/ABC/ACPM/ADA/AGS/APhA/ASPC/NLA/PCNA Guideline on the management of blood cholesterol. *J Am Coll Cardiol*. 2019;73(24):e285-e350. doi:10.1016/j.jacc.2018.11.003

48.	US Preventive Services Task Force, Davidson KW, Barry MJ, et al. Screening for colorectal cancer: US Preventive Services Task Force recommendation statement. *JAMA*. 2021;325(19):1965. doi:10.1001/jama.2021.6238

49.	Department of Veterans Affairs, Department of Defense. *VA/DoD Clinical Practice Guideline for the Management of Major Depressive Disorder*.; 2022. https://www.healthquality.va.gov/guidelines/MH/mdd/VADoDMDDCPGFinal508.pdf

50.	Liu Y, Collins C, Wang K, Bie R. The prevalence and trend of depression among veterans in the United States - ClinicalKey. *J Affect Disord*. 2019;245:724-727. doi:https://doi.org/10.1016/j.jad.2018.11.031

51.	Boulton AJM, Armstrong DG, Albert SF, et al. Comprehensive foot examination and risk assessment. *Diabetes Care*. 2008;31(8):1679-1685. doi:10.2337/dc08-9021

52.	Department of Veterans Affairs, Department of Defense. *VA/DoD Clinical Practice Guideline for the Management of Type 2 Diabetes Mellitus in Primary Care*.; 2017. https://www.healthquality.va.gov/guidelines/CD/diabetes/Vadoddmcpgfinal508.pdf

53.	Zhao H, McClure NS, Johnson JA, Soprovich A, Al Sayah F, Eurich DT. A longitudinal study on the association between diabetic foot disease and health-related quality of life in adults with type 2 diabetes. *Can J Diabetes*. 2020;44(3):280-286.e1. doi:10.1016/j.jcjd.2019.08.008

54.	American Diabetes Association Professional Practice Committee. Glycemic targets: Standards of medical care in diabetes—2022. *Diabetes Care*. 2022;45(Supplement_1):S83-S96. doi:10.2337/dc22-S006

55.	ElSayed NA, Aleppo G, Aroda VR, et al. Diabetes Technology: Standards of care in diabetes — 2023. *Diabetes Care*. 2023;46(Supplement_1):S111-S127. doi:10.2337/dc23-S007

56.	American Diabetes Association Professional Practice Committee, ElSayed NA, Aleppo G, et al. Chronic kidney disease and risk management: Standards of care in diabetes—2024. *Diabetes Care*. 2024;47(Supplement_1):S219-S230. doi:10.2337/dc24-S011

57.	Zingmond DS, Ettner SL, Wilber KH, Wenger NS. Association of claims-based quality of care measures with outcomes among community-dwelling vulnerable elders. *Med Care*. 2011;49(6):553-559. doi:10.1097/MLR.0b013e31820e5aab

58.	Gale MJ, Scruggs BA, Flaxel CJ. Diabetic eye disease: A review of screening and management recommendations. *Clin Experiment Ophthalmol*. 2021;49(2):128-145. doi:10.1111/ceo.13894

59.	American Diabetes Association Professional Practice Committee. Retinopathy, neuropathy, and foot care: Standards of medical care in diabetes—2022. *Diabetes Care*. 2022;45(Supplement_1):S185-S194. doi:10.2337/dc22-S012

60.	Oda G, Sharma A, Lucero-Obusan C, Schirmer P, Holodniy M. Exposure sources among Veterans with elevated blood lead levels, United States, 2015‒2021. *Am J Public Health*. 2022;112(S7):S670-S678. doi:10.2105/AJPH.2022.306936

61.	Department of Veterans Affairs, Department of Defense. *VA/DoD Clinical Practice Guideline for the Management of Posttraumatic Stress Disorder and Acute Stress Disorder*.; 2023. https://www.healthquality.va.gov/guidelines/MH/ptsd/VA-DoD-CPG-PTSD-Full-CPGAug242023.pdf

62.	Post-traumatic Stress Disorder by Gender and Veteran Status - ClinicalKey. Accessed February 19, 2025. https://www.clinicalkey.com/#!/content/playContent/1-s2.0-S0749379717304993?returnurl=https:%2F%2Flinkinghub.elsevier.com%2Fretrieve%2Fpii%2FS0749379717304993%3Fshowall%3Dtrue&referrer=https:%2F%2Fwww.frontiersin.org%2F

63.	Department of Veterans Affairs, Department of Defense. *VA/DoD Clinical Practice Guideline for Assessment and Management of Patients at Risk for Suicide*.; 2024. https://www.healthquality.va.gov/guidelines/MH/srb/VADOD-CPG-Suicide-Risk-Full-CPG-2024_Final_508.pdf

64.	Groeneveld PW, Medvedeva EL, Walker L, Segal AG, Menno DM, Epstein AJ. Association between spending and survival of chronic heart failure across Veterans Affairs Medical Centers. *JAMA Netw Open*. 2019;2(7):e197238. doi:10.1001/jamanetworkopen.2019.7238

65.	Heidenreich PA, Bozkurt B, Aguilar D, et al. 2022 AHA/ACC/HFSA Guideline for the management of heart failure: A report of the American College of Cardiology/American Heart Association Joint Committee on Clinical Practice Guidelines. *Circulation*. 2022;145(18). doi:10.1161/CIR.0000000000001063

66.	Yancy CW, Jessup M, Bozkurt B, et al. 2013 ACCF/AHA guideline for the management of heart failure: A report of the American College of Cardiology Foundation/American Heart Association Task Force on Practice Guidelines. *Circulation*. 2013;128(16). doi:10.1161/CIR.0b013e31829e8776

67.	Virani SS, Alonso A, Benjamin EJ, et al. Heart disease and stroke statistics—2020 update: A report from the American Heart Association. *Circulation*. 2020;141(9). doi:10.1161/CIR.0000000000000757

68.	Garren P, Serper M. Chronic hepatitis B in US Veterans. *Curr Hepatol Rep*. 2019;18(3):310-315. doi:10.1007/s11901-019-00479-9

69.	Sandul AL, Rapposelli K, Nyendak M, Kim M. Updated recommendation for universal hepatitis B vaccination in adults aged 19–59 years — United States, 2024. *MMWR Morb Mortal Wkly Rep*. 2024;73(48):1106. doi:10.15585/mmwr.mm7348a3

70.	Gonzalez R, Park A, Yakovchenko V, et al. HCV elimination in the US Department of Veterans Affairs. *Clin Liver Dis*. 2021;18(1):1-6. doi:10.1002/cld.1150

71.	Konerman MA, Beste LA, Van T, et al. Machine learning models to predict disease progression among veterans with hepatitis C virus. Bacciu D, ed. *PLOS ONE*. 2019;14(1):e0208141. doi:10.1371/journal.pone.0208141

72.	US Preventive Services Task Force, Owens DK, Davidson KW, et al. Screening for hepatitis C virus infection in adolescents and adults: US Preventive Services Task Force recommendation statement. *JAMA*. 2020;323(10):970. doi:10.1001/jama.2020.1123

73.	Chang ET, Zulman DM, Nelson KM, et al. Use of general primary care, specialized primary care, and other Veterans Affairs services among high-risk veterans. *JAMA Netw Open*. 2020;3(6):e208120. doi:10.1001/jamanetworkopen.2020.8120

74.	Sigel K, Park L, Justice A. HIV and cancer in the Veterans Health Administration System. *Semin Oncol*. 2019;46(4-5):334-340. doi:10.1053/j.seminoncol.2019.09.007

75.	US Preventive Services Task Force, Owens DK, Davidson KW, et al. Screening for HIV infection: US Preventive Services Task Force recommendation statement. *JAMA*. 2019;321(23):2326. doi:10.1001/jama.2019.6587

76.	Tsai J, Pietrzak RH, Szymkowiak D. The problem of Veteran homelessness: An update for the new decade. *Am J Prev Med*. 2021;60(6):774-780. doi:10.1016/j.amepre.2020.12.012

77.	Cohen AJ, Dosa DM, Rudolph JL, Halladay CW, Heisler M, Thomas KS. Risk factors for Veteran food insecurity: Findings from a National US Department of Veterans Affairs Food Insecurity Screener. *Public Health Nutr*. 2022;25(4):819-828. doi:10.1017/S1368980021004584

78.	McQuillan G, Kruszon-Moran D, Markowitz L, Unger E, Paulose-Ram R. *Prevalence of HPV in Adults Aged 18–69: United States, 2011–2014*.; 2017:8. Accessed February 19, 2025. https://www.cdc.gov/nchs/data/databriefs/db280.pdf

79.	Meites E, Szilagyi PG, Chesson HW, Unger ER, Romero JR, Markowitz LE. Human papillomavirus vaccination for adults: Updated tecommendations of the Advisory Committee on Immunization Practices. *MMWR Morb Mortal Wkly Rep*. 2019;68(32):698-702. doi:10.15585/mmwr.mm6832a3

80.	Centers for Disease Control and Prevention. *Flu Burden Prevented from Vaccination 2021-2022 Flu Season*.; 2023. https://www.cdc.gov/flu-burden/php/data-vis-vac/2021-2022-prevented.html

81.	Centers for Disease Control and Prevention. *Estimated Flu Disease Burden 2021-2022 Flu Season*.; 2024. https://www.cdc.gov/flu-burden/php/data-vis/2021-2022.html#:~:text=During%20the%202021%2D2022%20flu%20season%2C%20CDC%20estimates%20that%20flu,during%20the%202011%2D2012%20season.

82.	Grohskopf LA, Blanton LH, Ferdinands JM, Chung JR, Broder KR, Talbot HK. Prevention and control of seasonal influenza with vaccines: Recommendations of the Advisory Committee on Immunization Practices — United States, 2023–24 influenza season. *MMWR Recomm Rep*. 2023;72(2):1-25. doi:10.15585/mmwr.rr7202a1

83.	Marshall A, Panuzio J, Taft C. Intimate partner violence among military veterans and active duty servicemen. *Clin Psychol Rev*. 2005;25(7):862-876. doi:10.1016/j.cpr.2005.05.009

84.	US Preventive Services Task Force, Curry SJ, Krist AH, et al. Screening for intimate partner violence, elder abuse, and abuse of vulnerable adults: US Preventive Services Task Force final recommendation statement. *JAMA*. 2018;320(16):1678. doi:10.1001/jama.2018.14741

85.	Ju MR, Karalis JD, Chansard M, et al. Variation of hepatocellular carcinoma treatment patterns and survival across geographic regions in a Veteran population. *Ann Surg Oncol*. 2022;29(13):8413-8420. doi:10.1245/s10434-022-12390-7

86.	Marrero JA, Kulik LM, Sirlin CB, et al. Diagnosis, staging, and management of hepatocellular carcinoma: 2018 practice guidance by the American Association for the Study of Liver Diseases. *Hepatology*. 2018;68(2):723-750. doi:10.1002/hep.29913

87.	Singal AG, Zhang E, Narasimman M, et al. HCC surveillance improves early detection, curative treatment receipt, and survival in patients with cirrhosis: A meta-analysis. *J Hepatol*. 2022;77(1):128-139. doi:10.1016/j.jhep.2022.01.023

88.	Singal AG, Llovet JM, Yarchoan M, et al. AASLD Practice Guidance on prevention, diagnosis, and treatment of hepatocellular carcinoma. *Hepatology*. 2023;78(6):1922-1965. doi:10.1097/HEP.0000000000000466

89.	Zullig LL, Sims KJ, McNeil R, et al. Cancer incidence among patients of the U.S. Veterans Affairs Health Care System: 2010 update. *Mil Med*. 2017;182(7):e1883-e1891. doi:10.7205/MILMED-D-16-00371

90.	US Preventive Services Task Force. Screening for lung cancer: US Preventive Services Task Force recommendation statement. *JAMA*. 2021;325(10):962-970. doi:10.1001/jama.2021.1117

91.	Collins JP, Crowe SJ, Ortega-Sanchez IR, et al. Use of the Pfizer pentavalent meningococcal vaccine among persons aged ≥10 years: Recommendations of the Advisory Committee on Immunization Practices ― United States, 2023. *MMWR Morb Mortal Wkly Rep*. 2024;73(15):345-350. doi:10.15585/mmwr.mm7315a4

92.	MacNeil JR, Blain AE, Wang X, Cohn AC. Current epidemiology and trends in meningococcal disease—United States, 1996–2015. *Clin Infect Dis*. 2018;66(8):1276-1281. doi:10.1093/cid/cix993

93.	Mbaeyi S, Pondo T, Blain A, et al. Incidence of meningococcal disease before and after implementation of quadrivalent meningococcal conjugate vaccine in the United States. *JAMA Pediatr*. 2020;174(9):843-851. doi:10.1001/jamapediatrics.2020.1990

94.	Richmond BW, Miller RF. The Honoring Our PACT Act: An improved commitment to Veterans’ health. *Ann Am Thorac Soc*. 2023;20(4):508-509. doi:10.1513/AnnalsATS.202208-718VP

95.	Iraq and Afghanistan Veterans of America (IAVA). *10TH Annual IAVA Member Survey: Perceptions and Views of Iraq and Afghanistan Veterans on the Challenges and Successes of the next Greatest Generation of Veterans*.; 2020. https://iava.org/wp-content/uploads/2024/09/IAVA-2020-Member-Survey.pdf

96.	Obesity Medicine Association. Veterans affected by obesity. 2021. https://obesitymedicine.org/blog/obesity-in-veterans/

97.	Department of Veterans Affairs, Department of Defense. *VA/DoD Clinical Practice Guideline for the Management of Adult Overweight and Obesity*.; 2020. https://www.healthquality.va.gov/guidelines/CD/obesity/VADODObesityCPGFinal5087242020.pdf

98.	Department of Veterans Affairs. *Opioid Use Disorder*.; 2022. https://www.vacsp.research.va.gov/CSPEC/Studies/CSPEAR/Docs/Opioid-Use-Disorder.pdf

99.	US Preventive Services Task Force, Nicholson WK, Silverstein M, et al. Screening for osteoporosis to prevent fractures: US Preventive Services Task Force recommendation statement. *JAMA*. 2025;333(6):498. doi:10.1001/jama.2024.27154

100.	Sarafrazi N, Wambogo E, Shepherd J. *Osteoporosis or Low Bone Mass in Older Adults: United States, 2017–2018*.; 2021:8.

101.	Kobayashi M, Leidner AJ, Gierke R, et al. Use of 21-valent pneumococcal conjugate vaccine among U.S. adults: Recommendations of the Advisory Committee on Immunization Practices — United States, 2024. *MMWR Morb Mortal Wkly Rep*. 2024;73(36):793-798. doi:10.15585/mmwr.mm7336a3

102.	Mangen MJJ, Huijts SM, Bonten MJM, de Wit GA. The impact of community-acquired pneumonia on the health-related quality-of-life in elderly. *BMC Infect Dis*. 2017;17(1):208. doi:10.1186/s12879-017-2302-3

103.	Weycker D, Strutton D, Edelsberg J, Sato R, Jackson LA. Clinical and economic burden of pneumococcal disease in older US adults. *Vaccine*. 2010;28(31):4955-4960. doi:10.1016/j.vaccine.2010.05.030

104.	Lehavot K, Katon J, Chen J, Fortney J, Simpson T. Post-traumatic stress disorder by gender and Veteran status. *Clin Key*. 2018;54(1). doi:https://doi.org/10.1016/j.amepre.2017.09.008

105.	Department of Veterans Affairs, Department of Defense. *VA/DoD Clinical Practice Guideline for Management of Posttraumatic Stress Disorder and Acute Stress Disorder*.; 2023. https://www.healthquality.va.gov/guidelines/MH/ptsd/VA-DoD-CPG-PTSD-Full-CPG-Edited-11162024.pdf

106.	Wilson L. The prevalence of military sexual trauma: A meta-analysis. 2018. https://journals.sagepub.com/doi/full/10.1177/1524838016683459?journalCode=tvaa

107.	Havers FP, Moro PL, Hunter P, Hariri S, Bernstein H. Use of tetanus toxoid, reduced diphtheria toxoid, and acellular pertussis vaccines: Updated recommendations of the Advisory Committee on Immunization Practices — United States, 2019. *MMWR Morb Mortal Wkly Rep*. 2020;69(3):77-83. doi:10.15585/mmwr.mm6903a5

108.	Arnett DK, Blumenthal RS, Albert MA, et al. 2019 ACC/AHA guideline on the primary prevention of cardiovascular disease: A report of the American College of Cardiology/American Heart Association Task Force on clinical practice guidelines. *Circulation*. 2019;140(11). doi:10.1161/CIR.0000000000000678

109.	Meyers BM, Knox JJ, Cosby R, et al. Non-surgical management of advanced hepatocellular carcinoma: A systematic review by Cancer Care Ontario. *Can Liver J*. 2021;4(3):257-274. doi:10.3138/canlivj-2020-0039

110.	US Preventive Services Task Force, Krist AH, Davidson KW, et al. Interventions for tobacco smoking cessation in adults, Including pregnant persons: US Preventive Services Task Force recommendation statement. *JAMA*. 2021;325(3):265. doi:10.1001/jama.2020.25019

111.	Sun LY, Jones PM, Wijeysundera DN, Mamas MA, Bader Eddeen A, O’Connor J. Association between handover of anesthesiology care and 1-year mortality among adults undergoing cardiac surgery. *JAMA Netw Open*. 2022;5(2):e2148161. doi:10.1001/jamanetworkopen.2021.48161

112.	Department of Veterans Affairs, Department of Defense. *VA/DoD Clinical Practice Guideline for the Management and Rehabilitation of Post-Acute Mild Traumatic Brain Injury*.; 2021. https://www.healthquality.va.gov/guidelines/Rehab/mtbi/VADODmTBICPGFinal508.pdf

113.	Milsom I, Gyhagen M. The prevalence of urinary incontinence. *Climacteric*. 2019;22(3):217-222. doi:10.1080/13697137.2018.1543263
